# Updated list of Collembola species currently recorded from South Africa

**DOI:** 10.3897/zookeys.503.8966

**Published:** 2015-05-11

**Authors:** Charlene Janion-Scheepers, Louis Deharveng, Anne Bedos, Steven L. Chown

**Affiliations:** 1 Centre for Invasion Biology, Department of Botany and Zoology, Stellenbosch University, Private Bag X1, Matieland 7602, South Africa; 2 Institut de Systématique, Évolution, Biodiversité ISYEB - UMR 7205 - CNRS, MNHN, UPMC, EPHE, Muséum national d’Histoire naturelle, Sorbonne Universités, 45 rue Buffon, F-75005, Paris, France; 3 School of Biological Sciences, Monash University, Clayton, Victoria, Australia

**Keywords:** Biodiversity, endemicity, soil fauna, introduced species, taxonomy

## Abstract

Understanding the abundance and richness of species is one of the most fundamental steps in effecting their conservation. Despite global recognition of the significance of the below-ground component of diversity for ecosystem functioning, the soil remains a poorly studied terrestrial ecosystem. In South Africa, knowledge is increasing for a variety of soil faunal groups, but many still remain poorly understood. We have started to address this gap in the knowledge of South African soil biodiversity by focusing on the Collembola in an integrated project that encompasses systematics, barcoding and ecological assessments. Here we provide an updated list of the Collembola species from South Africa. A total of 124 species from 61 genera and 17 families has been recorded, of which 75 are considered endemic, 24 widespread, and 25 introduced. This total number of species excludes the 36 species we consider to be dubious. From the published data, Collembola species richness is high compared to other African countries, but low compared to European countries. This is largely a consequence of poor sampling in the African region, as our discovery of many new species in South Africa demonstrates. Our analyses also show that much ongoing work will be required before a reasonably comprehensive and spatially explicit picture of South Africa’s springtail fauna can be provided, which may well exceed 1000 species. Such work will be necessary to help South Africa meet its commitments to biodiversity conservation, especially in the context of the 2020 Aichi targets of the Convention on Biological Diversity.

## Introduction

The documentation of biodiversity is an essential first step for its conservation. A major barrier to so doing for invertebrates is a lack of taxonomic information on various groups. This taxonomic impediment and its implications for biodiversity studies have been widely discussed (Godfray 2002, Samper 2004). Despite these challenges, taxonomic knowledge continues to increase globally (Nilsson-Örtman and Nilsson 2010, Joppa et al. 2011, Platnick 2014, van Noort 2014). Nonetheless, given rapid environmental change and its effects on biodiversity (Butchart et al. 2010), it is unclear what the rate of extinction is for many groups (Pimm et al. 2010, Costello et al. 2013), complicating conservation efforts and assessments of their efficacy, thus underscoring the urgency to further document global biodiversity (Dirzo and Raven 2003, Bacher 2012).

This situation is as true for southern Africa as it is elsewhere. Knowledge of the South African fauna is increasing rapidly, especially in the case of a wide range of invertebrate groups (Foord et al. 2002, Robertson 2000, 2002, Parr et al. 2003, Dippenaar-Schoeman et al. 2006, Dippenaar-Schoeman and González Reyes 2006, Haddad and Dippenaar-Schoeman 2006, Hlavac 2007, Rousse and van Noort 2013). Nonetheless, many groups still remain relatively poorly studied, especially soil-dwelling taxa, which are essential for both above- and below-ground ecosystem functioning (Wardle et al. 2004, Hugo-Coetzee and Avenant 2011, Janion et al. 2011a). At the same time, considerable impacts on biodiversity continue to be documented as a consequence of habitat modification for agriculture and urban development, biological invasions, pollution, and climate change (Erasmus et al. 2002, Rouget et al. 2003, Biggs et al. 2008, Chown 2010, Pryke and Samways 2010, Huntley and Barnard 2012, Liu et al. 2012). In consequence, much need exists for documenting and understanding biodiversity and the processes underlying its variation across a wide range of groups, and especially the soil fauna.

Collembola are amongst the most widespread and abundant soil arthropods (Petersen and Luxton 1982, Hopkin 1997). Despite their obvious significance in soil systems, their utility as bioindicators (Lawrence 1953, Hopkin 1997, van Straalen 1998), their significance in the alien species faunas of many areas (Roques et al. 2009, Terauds et al. 2011), and the current growth in both morphological (Deharveng 2004) and molecular (Hogg and Hebert 2004, Rougerie et al. 2009) means to assess their diversity, they remain poorly known in South Africa. Indeed, by comparison with other regions of the world (Deharveng 2004), and other invertebrate taxa in the country (Scholtz and Chown 1995, Robertson 2000, Foord et al. 2011, Dippenaar-Schoeman 2014), knowledge of the group can be considered scanty.

The first attempt to collate all taxonomic information on the Collembola of South Africa was undertaken by Paclt (1959), listing *ca.* 65 species. Subsequently, an unpublished list entitled “Aquatic Collembola of South Africa” was made available online (P. Greenslade, no date), while Thibaud (2013) listed most publications until 2013. To date there are 38 publications on Collembola recorded or described from South Africa, the earliest by Börner (1908). Most notably, comprehensive descriptions were made by Yosii (1959), Paclt (1959, 1964, 1965, 1967), Coates (1968a, 1968b, 1969), Weiner and Najt (1991, 1998, 1999), and later Barra (1994, 1995, 1997, 1999, 2001, 2002, Barra and Weiner 2009). However, little other work has been done and the current list of species for the country is clearly an underestimate, with an incomplete understanding of which species might be introduced and thus might have substantial impacts, despite the fact that such impacts have been suggested for the country (Annecke and Moran 1982, Liu et al. 2012).

To address this substantial gap in the knowledge of soil biodiversity, a collaborative project was established in 2008 (Janion et al. 2011a, Bengtsson et al. 2011, 2012). Besides large-scale sampling and systematic assessments, which have resulted in new discoveries and species descriptions (Janion et al. 2011b, Potapov et al. 2011, Janion et al. 2012, 2013), a major component of the project has comprised the compilation of all currently available information on Collembola recorded from South Africa. Here we present this compilation as an updated checklist. It will provide a starting point for understanding the diversity of this group, as has been done for other geopolitical regions (e.g. Culik and Zeppelini 2003, Abrantes et al. 2010, 2012), and will assist South Africa to meet its obligations under the Convention on Biological Diversity (see for example Aichi Target 9 on identifying invasive alien species, and Aichi Target 17 on a national biodiversity strategy, http://www.cbd.int/sp/targets).

## Methods

All publications on Collembola species described or recorded from South Africa were collated from Salmon (1964) and Thibaud (2013). The list was checked and completed using the website “Checklist of the Collembola of the World” (Bellinger et al. 2014), the bi-annual bibliographical lists issued by the Museum National d’Histoire Naturelle (Paris, France), Zoological Record, Web of Science^TM^ (full date range of 1900 to 2014), and genus or species revisions from taxonomic journals sourced from the references identified using the original search methods. Nomenclature follows Bellinger et al. (2014), as it may have changed for certain taxonomic groups since the original description of the species. All published papers and webpages were examined and the following information was recorded when available: collection details including date, collector, province, place, nearest town, habitat type, and collection method, type locality and accession number if given. Only species with full species names were included in the species list of Table 2, thus excluding morphospecies identified to genus or to suspected species (e.g. *Seira* sp. or Isotomurus cf. maculatus). However, every record from the literature is listed in the Supplementary material (Suppl. material 1). The species were assigned a South African province from the locality recorded. From these points a species richness map was produced in ArcMap V10.2 (ESRI 2014).

The species were also divided into the following categories based on their distribution: 1) endemic if they were described from South Africa and have not been recorded elsewhere, 2) introduced if there is evidence from the literature that the species was introduced from another place, 3) widespread if the species is also present outside of South Africa but its origin is unknown, thus not considered introduced, or 4) dubious, when the species name given in the literature is considered a misidentification based on current taxonomic knowledge or if subsequent taxonomic work suggested this is the case (see Suppl. material 1).

To make an estimate of expected species richness, we used data collected from extensive sampling in the Western Cape Province of South Africa, which has been the main focus of much work on the group. The dataset comprises a total of 217 samples we obtained using several sampling techniques (see below) in as many localities and different microhabitats as possible throughout the Western Cape, including Afromontane forest, different fynbos vegetation types (see Mucina and Rutherford 2006), intertidal habitats, caves, and disturbed areas such as gardens and agricultural areas. Leaf litter, moss, rotten wood and soil samples were taken at different sampling sites over the duration of the project (2008–2012), and occasionally sieving and pitfall traps were also used. Typically, samples were extracted using a Berlese-Tullgren approach for five to seven days, or until dry (Berlese 1905, Tullgren 1918, Hopkin 1997). In addition, active searching was done in the field. Riparian soil was washed for water-dependent species, which were collected with a fine brush on the surface of water. Fine sand such as sea sand was washed in the laboratory and animals were also collected with a brush. Vegetation such as branches from bushes, fynbos shrubs, and grasses was beaten over a tray and animals were collected by means of an aspirator. All samples are stored in 96–99% ethanol at the Centre for Invasion Biology (C∙I∙B) Stellenbosch, or the Museum National d’Histoire Naturelle (MNHN) Paris. As identifications and species descriptions are still ongoing, we only used confirmed morphospecies for the purpose of calculating the number of species expected for the Western Cape.

Sampled-based rarefaction curves were plotted to estimate the number of species for the Western Cape, using Chao1 and Jacknife 2 in EstimateS V8.2.0 (Colwell 2009). Jacknife 2 does not require data to be normally distributed and provides conservative, but accurate estimates (Magurran 2004). Sampling is considered adequate when the rarefaction curves and the estimators converge at the highest observed values (Longino et al. 2002).

## Results

**Figure 1.**
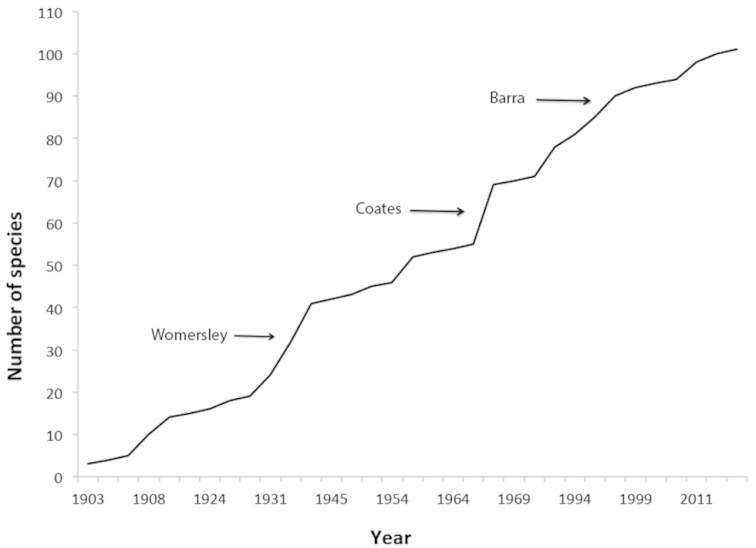
The cumulative number of Collembola species described from South Africa. The three major increases in described species are indicated by the author names (Womersley, Coates and Barra).

**Figure 2.**
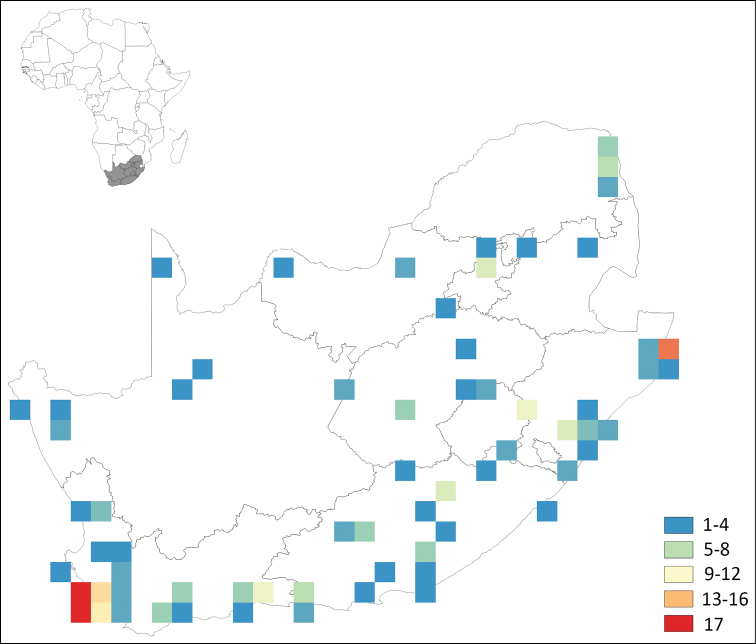
Number of Collembola species recorded for each degree square grid in South Africa.

According to the literature, a total of 160 species from 61 genera and 17 families have been recorded from South Africa (Table 1), with a relatively steady increase in descriptions since the first records in the early 1900s (Fig. 1). Of the recorded species, 36 are considered dubious, most of them misidentified records from Paclt (1959, 1967). Of the other species, 75 are endemic, 25 are thought to be alien species introduced to the country by human activity, and 24 have a widespread distribution, at least so far as current sampling indicates (Table 2). The majority of species have been recorded from the Western Cape (67 species), Kwazulu-Natal (46 species) and the Eastern Cape (20 species) (Fig. 2). Records from the other provinces are sparse (1–10 species), with the North West Province and Limpopo having the lowest recorded richness (three and one species, respectively). Although many authors did not indicate the habitat type where collections took place (Supplementary Material Suppl. material 1), the majority mentioned were from sites that are within the forest biome (see Mucina and Rutherford 2006 for full details of South Africa’s biomes and vegetation types). However, other vegetation types mentioned include those of the grassland biome and disturbed areas such as gardens, orchards and plantations.

**Table 1.** A summary of the Collembola species recorded from South Africa based on the literature.

	Number of species recorded from literature	Number of species accepted from literature	Introduced	Endemic	Widespread
**PODUROMORPHA**					
Hypogastruridae	19	11	4	5	2
Brachystomellidae	6	6	1	5	0
Neanuridae	16	15	2	10	3
Odontellidae	3	2	0	1	1
Onychiuridae	5	1	0	1	0
Tullbergiidae	8	7	1	3	3
**TOTAL**	**57**	**42**	**8**	**25**	**9**
**ENTOMOBRYOMORPHA**					
Isotomidae	23	19	5	8	6
Entomobryidae	49	36	8	25	3
Cyphoderidae	10	9	0	8	1
Paronellidae	1	1	0	0	1
Tomoceridae	1	1	0	1	0
TOTAL	**84**	**66**	**13**	**42**	**11**
**NEELIPLEONA**					
Neelidae	1	1	0	0	1
TOTAL	**1**	**1**	**0**	**0**	**1**
**SYMPHYPLEONA**					
Sminthurididae	2	1	0	0	1
Katiannidae	5	4	1	1	2
Dicyrtomidae	2	2	1	1	0
Bourletiellidae	7	7	1	6	0
Sminthuridae	2	1	1	0	0
TOTAL	**18**	**15**	**4**	**8**	**3**
**TOTAL**	**160**	**124**	**25**	**75**	**24**

**Figure 3.**
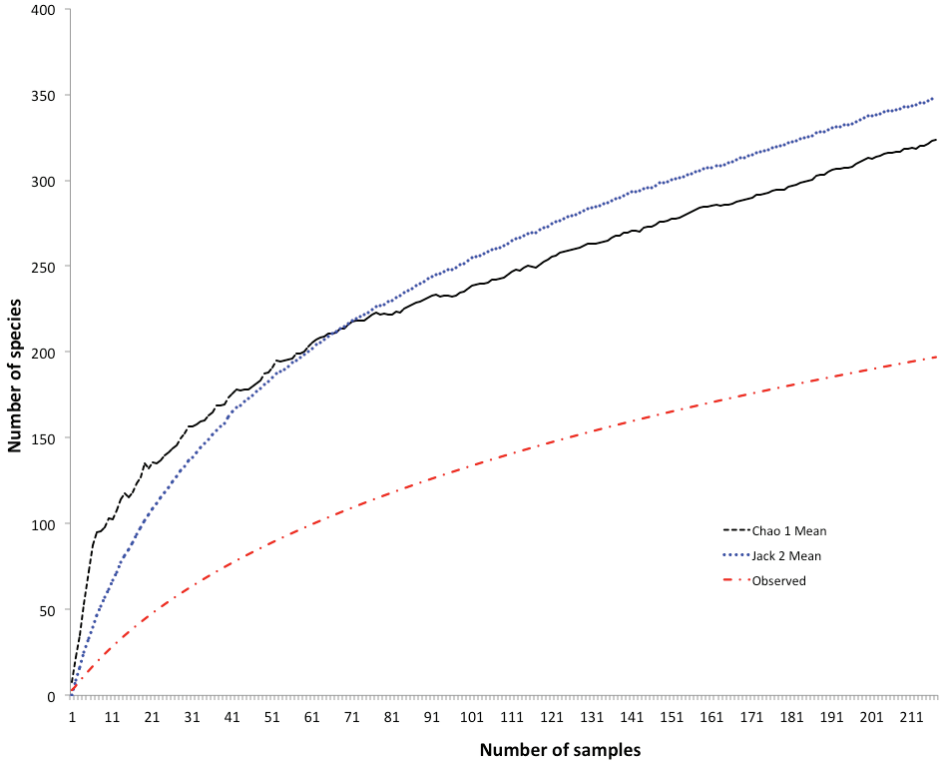
A sample-based rarefaction curve for the Western Cape, for observed species richness, and the Chao1 and Jacknife2 Estimators.

The sample-based species rarefaction curve for the Western Cape did not reach an asymptote (Fig. 3). The two richness estimators (Jacknife2: 348 species, Chao1 with 95% Confidence Intervals: 323, lower CI: 270, upper CI: 416) suggest that at least 6–7 times more than the number of species currently recorded from the literature will be found in the province, given the steep slope of the non-asymptotic curve.

**Table 2.** Collembola species recorded from South Africa, with “Current species name” as confirmed name (Bellinger et al. 2014), and “Name published in source” as name used in the original source when different from current species name. Abbreviations used: South Africa (SA), Western Cape (WC), Eastern Cape (EC), Kwazulu-Natal (KZN), Gauteng (G), Limpopo (L), Free State (FS), Northern Cape (NC), Mpumalanga (MP), North West Province (NWP), Lesotho (Les), endemic (E), introduced (I), dubious record (D) or widespread (W, species present outside of South Africa but not considered introduced). Genera endemic to South Africa are indicated by an asterisk (*). See Suppl. material 1: Table S1 for full collection and citation details.

Current species name	Source	Province recorded from in SA	Status	Habitat if given in source	Name published in source if different from the current one	Comments
**PODUROMORPHA**						
**Hypogastruridae**						
*Acherontiella thibaudi* Barra, 1994	Barra 1994	KZN	W	Beach sand		South Africa and several tropical regions of East Africa and Southeast Asia (Thibaud 2010)
*Austrogastrura lobata* (Yosii, 1959)	Yosii 1959	WC	E		*Choreutinula lobata* Yosii, 1959	
*Ceratophysella armata* (Nicolet, 1842)	Womersley 1934, Paclt 1959, 1967, Coates 1970	WC, KZN, EC, FS, G, NC	D	Damp soil, moss, litter	*Hypogastrura armata* Nicolet, 1842	Western palaearctic distribution.
*Ceratophysella armata trispina* (Womersley, 1934)	Womersley 1934	WC	D		Hypogastrura armata var. trispina Womersley, 1934	Described from a single specimen with three anal spines, could also have been *Triacanthella* sp.
*Ceratophysella longispina* (Tullberg, 1876)	Womersley 1934	NC, KZN,	D		*Hypogastrura longispina* Tullberg, 1876	Northern hemisphere circumpolar distribution (Fjellberg 1998)
*Hypogastrura manubrialis* (Tullberg, 1876)	Womersley 1934, Paclt 1959, 1967	NC, KZN, WC	I	Wet habitat		Distributed worldwide, considered introduced in the Southern hemisphere
*Hypogastrura manubrialis neglectus* (Börner, 1901)	Womersley 1934	WC	D		Hypogastrura manubrialis var. neglectus (Börner, 1901)	Dubious: lacks two anal spines, no more information provided.
*Hypogastrura purpurescens* (Lubbock, 1868)	Womersley 1934, Paclt 1959, 1967	WC	I	Wet leaves	*Hypogastrura pseudopurpurascens* Womersley, 1928 in Womersley 1934 Hypogastrura (Hypogastrura) purpurescens (Lubbock, 1868) in Paclt 1959, 1967	The species can be considered as introduced from Northern hemisphere, as has recently been confirmed for Australia (Greenslade et al. 2014).
*Hypogastrura sahlbergi* (Reuter, 1895)	Paclt 1959	WC	D	Near stream		Dubious record: holarctic distribution (Bellinger et al. 1996–2014).
*Hypogastrura sahlbergi rosea* (Reuter, 1895)	Womersley 1934	WC	D	Damp rocks	Hypogastrura sahlbergi var. rosea (Reuter, 1895)	Agrees with *sahlbergi* s. str. except for colour. *Species inquirenda*.
*Hypogastrura viatica* (Tullberg, 1872)	Womersley 1934, Paclt 1959	WC	I	Littoral		Nordic countries and Arctic, considered introduced in southern hemisphere (Greenslade 2002).
*Mesogastura libyca* (Caroli, 1914)	Paclt 1959	WC	D	Forest litter	*Choreutinula libyca* Caroli, 1914	Probably *Austrogastrura lobata* (Yosii, 1959), present in the same locality.
*Triacanthella madiba* Janion, D’Haese & Deharveng, 2012	Janion et al. 2012	WC	E	Cave guano		
*Willemia trilobata* Barra, 1995	Barra 1995	KZN	E	Beach sand		
*Xenylla capensis* Weiner & Najt, 1991	Weiner and Najt 1991	WC	E	Forest leaf litter		
*Xenylla maritima* Tullberg, 1869	Paclt 1959, 1967	WC, EC, KZN, NWP	I	Wet habitat, forest		Cosmopolitan distribution (Fjellberg 1998), probably introduced in the Southern hemisphere
*Xenylla rhodesiensis* Womersley, 1929	Coates 1970	MP	E	Wet habitat		
*Xenylla schillei* Börner, 1903	Paclt 1959	Les	D	At stream		Only recorded from Europe, while the collection locality in South Africa is very isolated and at a high altitude
*Xenylla yucatana* Mills, 1938	Barra 1995	KZN	W	Forest soil		Pan-tropical distribution (Deharveng et al. 2011)
**Brachystomellidae**						
*Brachystomella africana* Yosii, 1959	Yosii 1959	WC	E		*Brachystomella parvula africana* Yosii, 1959	
*Brachystomella coatesi* Weiner & Najt, 1991	Weiner and Najt 1991	WC	E	Forest leaf litter		
*Brachystomella georgensis* Weiner & Najt, 1991	Weiner and Najt 1991	WC	E	Forest leaf litter		
*Brachystomella parvula* (Schäffer, 1896)	Womersley 1934, Paclt 1959, 1967, Coates 1970	MP, WC, KZN, EC, FS	I	Wet litter		Cosmopolitan distribution (Fjellberg 1998)
*Probrachystomellides nicolaii* Weiner & Najt, 1991*	Weiner and Najt 1991	WC	E	Forest leaf litter		
*Setanodosa capitata* (Womersley, 1934)	Womersley 1934	WC	E		*Brachystomella capitata* Womersley, 1934	
**Neanuridae**						
*Aethiopella capensis* (Womersley, 1934)	Womersley 1934, Paclt 1959	WC, KZN	E	Stony stream	Ceratrimeria flavoantennatus var. capensis Womersley, 1934	
*Aethiopella handschini* (Denis, 1924)	Paclt 1959	Les, WC	D	Under stone, litter		Described and previously only known from Ethiopia (Massoud 1967)
*Anurida maritima* (Guérin-Méneville, 1836)	Womersley 1934, Paclt 1959, Yosii 1959, Lawrence 1953	WC, KZN	W	Littoral		Cosmopolitan distribution (Fjellberg 1998)
*Ectonura barrai* Janion, Bedos & Deharveng, 2011	Janion et al. 2011b	WC	E	Forest leaf litter		
*Ectonura coatesi* Barra, 1994	Barra 1994	KZN	E	Litter on dunes		
*Ectonura monochaeta* Janion, Bedos & Deharveng, 2011	Janion et al. 2011b	WC	E	Forest leaf litter		
*Ectonura natalensis* (Womersley, 934)	Womersley 1934, Paclt 1959	KZN, WC, EC	E	Litter	*Achorutes natalensis* Womersley, 1934 *Neanura natalensis* (Womersley, 1934)	
*Ectonura oribiensis* (Coates, 1968)	Coates 1968	KZN	E	Soil, litter	*Neanura oribiensis* Coates, 1968	
*Friesea claviseta* Axelson, 1900	Womersley 1934	KZN, WC	I	Litter		Cosmopolitan, possibly introduced in the southern hemisphere
*Friesea versabilis* Barra, 1995	Barra 1995	KZN	W	Under vegetation		Recorded from South Africa and Madagascar (Thibaud 2008)
*Najtafrica riebi* (Barra, 1994)*	Barra 1994	KZN	E	Dune litter	*Stachorutes riebi* Barra, 1994	
*Neanura muscorum* (Templeton, 1835)	Coates 1968a	EC	I	Litter		Sub-cosmopolitan, introduced in the southern hemisphere. All other species of the genus are in Europe.
*Pseudachorutella africana* Weiner & Najt, 1991	Weiner and Najt 1991	WC	E	Forest leaf litter		
*Pseudachorutes alluaudi* (Delamare Deboutteville, 1946)	Paclt 1959	KZN	W	Forest leaf litter	*Ceratrimeria alluaudi* Delamare Deboutteville, 1946	Described and only known so far from Eastern Africa (Massoud 1967).
*Pseudachorutes univesicatus* Weiner & Najt, 1991	Weiner and Najt 1991	WC	E	Forest leaf litter		
*Vitronura joanna* (Coates, 1968)	Coates 1968a	NWP	E	Soil	*Neanura joanna* Coates, 1968	
**Odontellidae**						
*Odontella sylvatica* Weiner & Najt, 1991	Weiner and Najt 1991	WC	E	Forest leaf litter		
*Odontellina deharvengi* Barra, 1995	Barra 1995	KZN	W	Soil		Recorded from South Africa and Madagascar (Thibaud 2008)
*Superodontella empodialis* (Stach, 1934)	Paclt 1959	KZN	D		*Odontella empodialis* Stach, 1934	Dubious identification, European distribution
**Onychiuridae**						
*Deuteraphorura inermis* (Tullberg, 1869)	Womersley 1934, Paclt 1959	WC	D	Under stones	*Onychiurus fimetarius* (Linné, Lubbock) (sic) in Womersley 1934 *Onychiurus pseudinermis* Börner, Börner 1903 in Paclt 1959	Given the confusion around the species *fimetarius*, *inermis* and *pseudinermis*, and the age of the specimen slides, the identification given by authors (following Bellinger et al. 1996-2014) is uncertain.
*Orthonychiurus camerunensis* (Schött, 1926)	Paclt 1967	G	D	Soil	*Onychiurus camerunensis* Schött, 1926	The Schött description is insufficient to recognize the species.
*Orthonychiurus saasveldensis* (Weiner & Najt, 1991)	Weiner and Najt 1991	WC	E	Forest, on bark	*Onychiurus saasveldensis* Weiner & Najt, 1991	
*Protaphorura armata* (Tullberg, 1869)	Lawrence 1953	?	D		*Onychiurus armatus*	A holarctic distribution. Southern records of *Protaphorura* are usually *Thalassaphorura* species, or possible introductions.
*Protaphorura matsumotoi* (Kinoshita, 1923)	Paclt 1959	FS	D	Soil	*Onychiurus matsumotoi* Kinoshita, 1923	A *species inquirenda* after Yosii (1977), only recorded so far from Japan.
**Tullbergiidae**						
*Delamarephorura capensis* Janion, Weiner & Deharveng, 2013	Janion et al. 2013	WC	E	Soil		
*Delamarephorura szeptyckii* Barra & Weiner, 2009	Barra and Weiner 2009	EC	E	Dry grassland		
*Fissuraphorura miscellanea* Barra, 1995	Barra 1995	KZN	E	Soil		
*Mesaphorura krausbaueri* (Börner, 1901)	Womersley 1934, Paclt 1959	WC, EC, FS	D	Soil, under stones	*Tullbergia krausbaueri* Börner, 1901	Dubious identification, most *Mesaphorura* have been identified as *Mesaphorura krausbaueri* before the split of this species by Rusek (1971). Older records are not reliable (Fjellberg 1998).
*Mesaphorura yosii* (Rusek, 1967)	Barra 1995	KZN	W			Cosmopolitan distribution
*Paratullbergia callipygos* (Börner, 1902)	Womersley 1934	WC	I		*Tullbergia callipygos* Börner, 1902	Holarctic distribution
*Tullbergia meridionalis* Cassagnau & Rapoport, 1962	Barra 1995	KZN	W	Dune sand		Described from Argentina and later recorded from South Africa.
*Tullbergia kilimanjarica* (Delamare Deboutteville, 1953)	Paclt 1959, 1967, Coates 1970	WC, KZN, MP	W	Forest leaf litter, garden soil	*Mesaphorura kilimanjarica* Delamare Deboutteville, 1953	Described from Tanzania and later recorded from South Africa.
**ENTOMOBRYOMORPHA**						
**Isotomidae**						
*Archisotoma sabulosa* Barra, 1997	Barra 1997	KZN	E	Littoral dune sand		
*Arlea tridens* Barra, 1997	Barra 1997	KZN	E	Dune litter		
*Ballistura schoetti* (Dalla Torre, 1895)	Womersley 1934, Yosii 1959, Paclt 1959, 1967	WC, EC	I	Vegetation, rain pools	*Proisotoma schoetti* (Dalla Torre, 1895) in Womersley 1934 and Paclt 1959, 1967	Cosmopolitan distribution
*Clavisotoma africana* (Womersley, 1934)	Womersley 1934, Paclt 1959	WC	E	Wet leaves, rain pools	*Proisotoma africana* (Womersley, 1934)	
*Folsomides americanus* Denis, 1931	Paclt 1959, Barra 1997	KZN	W	From dry leaves		Cosmopolitan distribution
*Folsomina onychiurina* Denis, 1931	Barra 1997	KZN	W			Pantropical distribution
*Hemisotoma thermophila* (Axelson, 1900)	Womersley 1934, Paclt 1959, Coates 1970	KZN, WC	W	Under rotting leaves	*Isotoma bituberculata* Wahlgren, 1906 in Womersley 1934 and in Paclt 1959 *Isotomina thermophila* (Axelson, 1900) in Coates 1970	Cosmopolitan distribution. *Isotoma bituberculata* is proposed as a synonym of either *Hemisotoma thermophila* or *Hemisotoma orientalis* (Stach, 1947) in Potapov (2001). We provisionally consider it as a synonym of *Hemisotoma thermophila*, the most widespread species of the genus *Hemisotoma*.
*Isotoma finitima* Scherbakov, 1899	Paclt 1959	KZN	D		*Sorensia finitima* (Scherbakov, 1899)	The species is described without PAO, but body pigment is present; as such, it does not fit any known genus (Potapov 2001). Species inquirenda.
*Isotoma mauretanica* Handschin, 1926	Womersley 1934	WC	D			*Species inquirenda*. Stach (1947) considered this Algerian species as a possible member of the genus *Isotomurus*, but the description is too brief to support such a statement. See also *Isotomurus palustris*.
*Isotomiella sodwana* Barra, 1997	Barra 1997	KZN	E	Litter and humus on sand dunes		
*Isotomodes productus* (Axelson, 1906)	Womersley 1934	WC	I	Under stones		Subcosmopolitan, records from southern hemisphere scattered.
*Isotomurus balteatus* (Reuter, 1876)	Womersley 1934	WC	D		Isotomurus palustris var. balteata (Reuter, 1876).	*Isotomurus balteatus* is a species of Europe recognizable by its transversal stripes on tergites. We have seen such a colour pattern in South African Isotomidae of an undetermined genus which is not *Isotomurus*. The record of this species for South Africa is therefore dubious.
*Isotomurus palustris* (Müller, 1776)	Womersley 1934, Paclt 1959, 1967	WC, EC, G, KZN	I			Specimens of *Isotoma mauretanica* Handschin, 1926 recorded in Womersley 1934 were re-identified as *Isotomurus palustris* by Paclt (1959). This change is probably wrong, as Paclt states that specimens lack bothriotrichia.
*Isotomurus tricuspis* Börner, 1906	Paclt 1959, 1967	WC	D	Damp moss		Specimens of Isotomurus palustris var. balteata cited by Womersley (1934) are considered as *Isotomurus tricuspis* by Paclt (1959), based on similar pattern. of transversal stripes on tergites. However, these South African forms need to be examined morphologically to confirm their congeneric status with I. tricuspis from Java.
*Micranurophorus musci* Bernard, 1977	Barra 1997	KZN	W	Humid sand 20 cm under pioneer vegetation		Subcosmopolitan interstitial species.
*Mucrosomia caeca* (Wahlgren, 1906)	Paclt 1959	KZN, WC	W	From wet debris	*Cryptopygus caecus* Wahlgren, 1906	Current name after Potapov (2001).
*Parisotoma mossopi* (Womersley, 1934)	Paclt 1959	FS	E	From soil containing organic material	Isotoma notabilis ssp. mossopi Womersley, 1934	
*Parisotoma notabilis* (Schäffer, 1896)	Paclt 1959, 1967	WC	I	Wet leaves, leaf litter,	*Isotoma notabilis* Schäffer, 1896 in Paclt 1959, 1967	
*Parisotoma obscurocellata* Potapov, Janion & Deharveng, 2011	Potapov et al. 2011	WC	E	Litter under plants, coastal		
*Parisotoma sexsetosa* Potapov, Janion & Deharveng, 2011	Potapov et al. 2011	WC	E	Forest leaf litter		
*Pauropygus caussaneli* (Thibaud, 1996)	Barra 1997	KZN	W	Littoral sand	*Cryptopygus riebi* Barra, 1997	Synonymy after Potapov, Gao and Deharveng 2013. On the coasts of Indian and Atlantic Oceans
*Proisotoma davidi* Barra, 2001	Barra 2001	EC	E	Grassland soil		
*Proisotoma minuta* (Tullberg, 1871)	Paclt 1959, 1967	WC, KZN, FS, EC	I	Litter		Cosmopolitan species.
**Entomobryidae**						
*Capbrya marshalli* Barra, 1999*	Barra 1999	EC	E	Grassland		
*Capbrya themeda* Barra, 1999*	Barra 1999	EC	E	Grassland		
*Coecobrya caeca* (Schött, 1896)	Goto 1953	WC	D	In cave	*Sinella coeca* (Schött, 1896)	*Coecobrya coeca* is restricted to northern America according to Chen and Christiansen (1997), and unlikely to have been introduced in South African caves. The South African species might be the cosmopolitan *Coecobrya tenebricosa* (Folsom, 1902) (Zhang et al. 2009)
*Coecobrya hoefti* (Schäffer, 1896)	Paclt 1959	WC	D	In cave		Extra-European records are dubious (Jordana 2012). The Paclt specimens, from the same locality as the Goto (1953) specimens, may rather belong to the cosmopolitan species *Coecobrya tenebricosa* (Zhang et al. 2009)
*Entomobrya atrocincta* Schött, 1897	Paclt 1967	WC	I?	Litter		The large distribution of the species makes it difficult to determine from which region it may have been introduced. In addition, most colour patterns described in the literature do not fit the original and clear description of Schött (1897).
*Entomobrya decemfasciata* (Packard, 1873)	Womersley 1934	WC	D			Contrary to the claim of Womersley, *Entomobrya decemfasciata* does not occur in “most temperate parts of the world, including Europe”. Reliable records are restricted to North America. The colour pattern given by Womersley is different from that given by Christiansen and Bellinger (1998) for specimens of the USA.
*Entomobrya lanuginosa* (Nicolet, 1842)	Womersley 1934	WC	I?		Entomobrya nivalis Linnaeus, 1758 f. immaculata Schäffer, 1896	The cited form is tentatively reported to *Entomobrya lanuginosa*. In that case it would be an introduced species.
*Entomobrya minima* Brown, 1926	Brown 1926	KZN	E	Under stone		
*Entomobrya multifasciata* (Tullberg, 1871)	Paclt 1967	WC, NC, G	I	Litter, next to stream		Widespread in the holarctic region.
*Entomobrya nicoleti* (Lubbock, 1876)	Womersley 1934	WC	I?		Entomobrya nivalis f. maculata Schäffer, 1896	The cited form is tentatively reported to *Entomobrya nicoleti*. In that case it would be an introduced species.
*Entomobrya nivalis* (Linnaeus, 1758)	Paclt 1959, 1967, Coates 1970	WC, EC, FS, KZN	I	Litter, rainwater pool		Cosmopolitan distribution, but most reliable records are in the holarctic region.
*Lepidocyrtus cyaneus* Tullberg, 1871	Paclt 1959	KZN, EC	I?	Dry leaves, damp soil		Cosmopolitan distribution, but considered introduced in southern hemisphere where other related species are absent.
*Lepidocyrtus ferrugineus* (Schött, 1893)	Paclt 1959	KZN	D	Dry leaves		Described from Africa, the species needs a modern redescription to be recognizable.
*Lepidocyrtus lanuginosus* (Gmelin, 1788)	Womersley 1934, Paclt 1967	WC	D	Litter		Records of this species from the southern hemisphere need to be checked.
*Lepidokrugeria meyerae* Coates, 1969*	Coates 1969	MP	E	Dead leaves		
*Orchesella hexfasciata* (Harvey, 1896)	Paclt 1959	FS, G	D	Litter	*Entomobrya hexfasciata* Harvey, 1896	Assigned to the genus *Entomobrya* by Paclt (1959), today considered as an *Orchesella* (Christiansen and Bellinger 1998). All reliable records are from the USA.
*Pseudosinella alba* (Packard, 1873)	Paclt 1959	WC, EC	I	Litter		Cosmopolitan distribution, but most reliable records are in the holarctic region.
*Pseudosinella biguttata* Barra 1997	Barra 1997	KZN	E	Sand forest litter		
*Pseudosinella immaculata* (Lie–Pettersen, 1897)	Paclt 1959	KZN	D			All reliable records of this species are from Western Europe (Gisin and Da Gama 1972), following major taxonomic changes in species delimitations introduced in the 60’
*Pseudosinella octopunctata* Börner, 1901	Paclt 1959	WC, FS	I?	Wet litter		Subcosmopolitan distribution, but most tropical and southern hemisphere records need confirmation.
*Seira addoensis* Coates, 1968	Coates 1968	EC	E	Soil and vegetation		
*Seira anncla* Coates, 1968	Coates 1968, 1970	EC, WC	E	Shore vegetation		
*Seira annulicornis* (Börner, 1903)	Yosii 1959, Coates 1968, 1970	WC, MP, G, FS, KZN,	W		Seira (Lepidocyrtinus) annulicornis (Börner, 1903) in Yosii 1959	African distribution
*Seira annulipes* (Handschin, 1929)	Womersley 1934	KZN, WC	W	On vegetation	*Lepidocyrtus annulipes*, mispelling for *Lepidocyrtinus annulipes* Handschin, 1929	African distribution. Redescription needed on modern *standards*.
*Seira annulosa* (Wahlgren, 1906)	Womersley 1934	WC	D	Shore vegetation	Lepidocyrtinus flavovirens var. annulosa Wahlgren, 1906	Species previously known from Sudan; morphological features given by Wahlgren and Womersley do not allow reliable identification.
*Seira barnardi* (Womersley, 1934)	Womersley 1934, Yosii 1959, Paclt 1959, 1967, Coates 1968, 1970	WC, NWP	E	Wet leaves	Lepidocyrtinus cooperi var. barnardi Womersley, 1934 Seira (Lepidocyrtinus) barnardi (Womersley, 1933) (sic)	
*Seira capensis* (Womersley, 1934)	Womersley 1934, Yosii 1959, Coates 1968	WC, EC	E	On vegetation	*Lepidocyrtinus capensis* Womersley, 1934 Seira (Seira) capensis (Womersley, 1934) in Yosii 1959	
*Seira damerella* Coates, 1968	Coates 1968, 1970	L, MP	E	Litter		
*Seira dayi* Yosii, 1959	Yosii 1959, Coates 1968	WC	E		Seira (Lepidocyrtinus) dayi Yosii, 1959	
*Seira eleana* Coates, 1968	Coates 1968, 1970	MP	W	From dry vegetation		Also recorded from Mozambique by Coates (1968) and from Yemen by Barra (2004)
*Seira flavovirens* (Börner, 1903)	Womersley 1934, Yosii 1959, Coates 1968	WC	D		*Lepidocyrtinus flavovirens* Börner, 1903 in Womersley 1934; author should be (Börner, 1903) Seira (Seira) flavovirens (Börner, 1903) in Yosii 1959	May correspond to several whitish species of *Seira*.
*Seira grisea* (Womersley, 1934)	Womersley 1934, Coates 1968	WC	E	From vegetation	*Pseudosira grisea* Womersley, 1934	Possibly a synonym of *Seira flavovirens* according to Yosii (1959)
*Seira grisea annulata* (Womersley, 1934)	Womersley 1934	WC	D		Pseudosira grisea var. annulata Womersley, 1934	The taxonomic value of this form is uncertain. This variety might be synonym of *Seira flavovirens* after Yosii (1959).
*Seira incerta* (Handschin, 1926)	Womersley 1934	WC	D	Estuary	*Lepidocyrtinus incertus* Handschin, 1926	The species has a characteristic colouration, but is only known from the Mediterranean region where it is uncommon, so unlikely to have been introduced to South Africa.
*Seira laeta* (Börner, 1908)	Börner 1908	NC	E		Pseudosira (Mesira) laeta Börner, 1908	
*Seira lindei* Coates, 1968	Coates 1968	EC, WC	E	Wet litter		
*Seira marephila* Coates, 1968	Coates 1968	EC, WC	E	Litter		
*Seira mathewsi* Coates, 1968	Coates 1968, 1970	EC, WC	E	From vegetation		
*Seira metala* Coates, 1968	Coates 1968	WC	E	Litter		
*Seira metarsiosa* Coates, 1968	Coates 1968	FS, NC	E	From grass		
*Seira munroi* (Paclt, 1959)	Paclt 1959	NC	E	In ants’ nest	*Diamantinum munroi* Paclt, 1959	Transferred to *Seira* by Salmon (1964)
*Seira nagatai* Yosii, 1959	Yosii 1959	WC	E		Seira (Seira) nagatai Yosii, 1959	
*Seira pallens* (Börner, 1908)	Börner 1908	NC	E		Pseudosira nyassica var. pallens Börner, 1908	
*Seira pseudocoerulea* (Denis, 1924)	Womersley 1934, Yosii 1959	WC	D	Estuary	*Lepidocyrtinus pseudocoeruleus* (Denis, 1924) in Womersley 1934	African species. A study of the chaetotaxy of Ethiopian specimens would be however necessary to confirm identification (Yosii 1959).
*Seira rowani* Yosii, 1959	Yosii 1959, Coates 1968, 1970	WC	E	On vegetation	Seira (Afroseira) rowani Yosii, 1959	
*Seira rykei* Coates, 1968	Coates 1968	WC	E	On vegetation		
*Seira squamoornata* (Scherbakov, 1898)	Paclt 1959, 1967	KZN, WC, FS, G, NC	D	Soil and vegetation		The numerous records of this species by Paclt are all dubious, and concern various endemic species of the genus. *Seira squamoornata* is today considered to be limited to the Palaearctic region.
*Seira tsikama* Coates, 1968	Coates 1968, 1970	WC	E	Forest leaf litter		
*Seira vaneedeni* Coates, 1968	Coates 1968	KZN	E	From shrub and grass		
**Cyphoderidae**						
*Calobatinus rhadinopus* (Börner, 1913)	Börner 1913, Paclt 1967	KZN, G	E	Termite nest	*Calobatella rhadinopus* Börner, 1913	
*Cyphoda colura* (Börner, 1908)	Börner 1908	NC	E	Termite nest	*Cyphoderus colurus* Börner, 1908	
*Cyphoda limboxiphia* (Börner, 1913)	Börner 1913, Paclt 1967	KZN, G	E?	Termite nest	*Cyphoderus limboxiphius* Börner, 1913	
*Cyphoda natalensis* (Börner, 1913)	Börner 1913, Womersley 1934	KZN, WC	E	Termite nest	*Cyphoderus natalensis* Börner, 1913	
*Cyphoderus assimilis* (Börner, 1906)	Paclt 1959	KZN	W	Ant nest		Cosmopolitan distribution
*Cyphoderus bidenticulatus* Parona, 1888	Börner 1913	KZN	E	Termite nest		
*Cyphoderus omoensis* Delamare Deboutteville, 1945	Paclt 1959, Womersley 1934	WC	D	In cave	Cyphoderus arcuatus var. aethiopicus Hanschin, 1929 in Womersley 1934,	Wrong identification of Womersley after Paclt (1959)
*Cyphoderus squamidives* Silvestri, 1918	Silvestri 1918, Paclt 1959, 1967	KZN, WC, G	E?	Termite nest	Cyphoderus arcuatus var. squamidives in Silvestri 1918	
*Cyphoderus trinervoidis* Paclt, 1965	Paclt 1965	G	E	Termite nest		
*Pseudocyphoderus wasmanni* Börner, 1913	Börner 1913, Paclt 1967	KZN, G	E	Termite nest		
**Paronellidae**						
*Dicranocentruga nigromaculata* (Schött, 1903)	Paclt 1959	KZN	W		*Paronella nigromaculata* Schött, 1903 in Paclt 1959	African species. The generic name *Dicranocentruga* Wray, 1953 was reactivated by Mitra (2002)
**Tomoceridae**						
*Neophorella dubia* Womersley, 1934*	Womersley 1934	WC	E			
**NEELIPLEONA**						
**Neelidae**						
*Megalothorax minimus* (Willem, 1900)	Paclt 1967	WC	W	Damp soil, moss		Cosmopolitan species, currently in course of splitting. South Africa specimens will have to be re-examined.
**SYMPHYPLEONA**						
**Sminthurididae**						
*Denisiella serroseta* (Börner, 1908)	Börner 1908, Paclt 1959	NC	W		Sminthurides (Stenacidia) serroseta in Börner 1908; Sminthurides (Denisiella) serroseta in Paclt 1959	African species
*Sphaeridia minima* (Schött, 1893)	Paclt 1959, 1967	FS, WC	D	From soil	Sminthurides (Sphaeridia) minimus (Schött, 1893)	*Sphaeridia minima* is distributed in western Africa. It is very similar, if not identical, to the cosmopolitan species *Sminthurides pumilis* Krausbauer, 1898. Bretfeld (1999) considers that the Paclt specimens may belong to *Sminthurides pumilis*, but that those from Cameroon may represent distinct species. A revision of these tropical *Sphaeridia* is clearly needed.
**Katiannidae**						
*Katianna kerguelenensis* Denis, 1947	Paclt 1959	KZN	D			The South African records of this sub-Antarctic species need confirmation.
*Sminthurinus mime* (Börner, 1907)	Womersley 1931, Paclt 1959, Paclt 1967	WC	W	Beneath vegetation	*Sminthurinus terrestris* Womersley, 1931	Paclt (1959) mentions differences between the two species, that are nevertheless synonymized by Greenslade (1994). Widely distributed in the southern hemisphere and in tropical Asia.
*Sminthurinus niger* (Lubbock, 1873)	Womersley 1931, Paclt 1959	WC	I	Under loose bark		Mostly holarctic. Tropical and Australian records may be the result of introductions.
*Sminthurinus pallidus* Womersley, 1931	Womersley 1931, Paclt 1959	WC	E	Beneath vegetation	*Sminthurinus terrestris* Womersley, 1931 in Paclt 1959	The synonymy of *Sminthurinus pallidus* Womersley 1931 with *Sminthurinus terrestris* proposed by Paclt (1959) is based on unsufficient ground and not accepted here.
*Stenognathellus stenognathus* (Börner, 1907)	Paclt 1959	WC, KZN	W	Litter	*Sminthurinus stenognathus* (Börner, 1907)	Africa and Argentina.
**Dicyrtomidae**						
*Dicyrtomina africana* Womersley, 1931	Womersley 1931	WC	E	On vegetation	Dicyrtomina minuta form africana Womersley, 1931	The validity of this form needs confirmation.
*Dicyrtomina minuta* (O. Fabricius, 1783)	Paclt 1959, 1967	WC	I	At stream, on vegetation		Northern hemisphere, probably introduced in southern regions. Paclt considered Dicyrtoma minuta f. africana as identical with *Dicyrtomina minuta*.
**Bourletiellidae**						
*Bourletiella arvalis* (Fitch, 1863)	Paclt 1959	WC	I	Lucerne pasture	Bourletiella (Bourletiella) arvalis (Fitch, 1863)	Northern hemisphere, with local occurrence in southern hemisphere where it has been probably introduced.
*Prorastriopes barnardi* (Womersley, 1931)	Womersley 1931, Paclt 1959	WC	E	Amongst grass	Deuterosminthurus marmoratus var. barnardi Womersley, 1931	A colour form of *Prorastriopes marmoratus*. Paclt (1959) synonymized this form with *Rastriopes schultzei* on insufficient evidence.
*Prorastriopes marmoratus* (Womersley, 1931)	Womersley 1931, Paclt 1959	WC	E	Rainwater pools	*Deuterosminthurus marmoratus* Womersley, 1931	Paclt (1959) synonymized this species with *Rastriopes schultzei* on insufficient evidence.
*Prorastriopes schultzei* (Börner, 1908)	Börner 1908	WC, G, NC	E	Among vegetation, wet habitat	*Bourletiella schultzei* in Börner, 1908	Generic assignation after Betsch (1980). Paclt (1959) proposes to synonymize *Prorastriopes marmoratus*, *Prorastriopes banardi* and *Prorastriopes schultzei*, with *Rastriopes lineata* on weak morphological evidence as all these species are too briefly described. The same author considers in 1967 that his previous citation of *schultzei* (in Paclt 1959) as *Rastriopes lineatus* (here *Rastriopes lineata*).
*Prorastriopes webbi* Paclt, 1964	Paclt 1964, Coates 1970	KZN, MP, EC	E	On vegetation, litter		
*Rastriopes lineata* Womersley, 1931	Womersley 1931, Paclt 1959, 1967	WC, NC, G	E	Under a fallen twig and on rainwater pool (Womersley 1931), on vegetation (Paclt 1959), moss and rotten leaves, grass on river banks (Paclt 1967)	*Rastriopes schultzei* in Paclt 1959	Paclt (1959) synonymized this species with *Rastriopes schultzei*, but in 1967 considered that the specimen he identified as *schultzei* in Paclt (1959) was in fact *Rastriopes lineata*, bona species.
*Tritosminthurus schuhi* Snider, 1988*	Snider 1988	WC	E			
**Sminthuridae**						
*Papirinus prodigiosum* Yosii, 1954	Paclt 1959	KZN	D		*Sphyrotheca prodigiosa* (Yosii, 1954)	The genus *Papirinus*, placed among Katiannidae in Bretfeld (1999), is considered here as closer to Sminthuridae. This species is only known from Japan. Other species exist in Madagascar, Sumatra, Thailand and Congo. The South African species is probably new (Betsch 1980).
*Sminthurus viridis* (Linnaeus, 1758)	Lawrence 1953, Paclt 1959	WC	I	On vegetation		Mainly holarctic species, thought to have been introduced from Europe (via Australia) as eggs in soil through the importation of clover seed (Wallace 1968, Wallace and Walters 1974).

## Discussion

The number of Collembola species recorded for South Africa is low compared to well-studied regions such as Europe (Deharveng 2007), but is the highest of all African countries south of Sahara (Thibaud 2013). Low sampling intensity in Africa seems to be the main reason for this pattern. Based on new records and species discovered during recent systematic sampling in the Western Cape Province alone (Janion et al. 2011a, b, Potapov et al. 2011, Janion et al. 2012, Liu et al. 2012, Janion et al. 2013), it is clear that many species remain to be recorded and described for this province. Given low richness documented elsewhere in South Africa the same situation is likely to be the case both there and in other African countries. The spatial distribution of species richness records also suggests that incomplete sampling coverage lies at the heart of the current diversity patterns. Most records to date have come from those provinces where taxonomists were either based or hosted such as in Cape Town of the Western Cape Province (Womersley 1934, Paclt 1959, Yosii 1959), and in Pretoria of the Gauteng Province (Coates 1969), reflecting a recurrent bias in geographic patterns of diversity of poorly known groups (Deharveng et al. 2000). Although Collembola do generally prefer moist environments (Hopkin 1997), which may mean lower diversity in arid provinces such as the Northern Cape and North-West Provinces (see Mucina and Rutherford 2006), low species richness in provinces such as Limpopo and Kwazulu-Natal is at odds with most other groups in the country (see e.g. Davis 1997 for dung beetles, Erasmus et al. 2000 for antlions, Foord et al. 2002 for spiders, Evans et al. 2006 for amphibians and birds, Schoeman and Foord 2012 for ants). The only exception to the poor knowledge of the fauna is for the sub-Antarctic Prince Edward Island group (consisting of Marion Island and the smaller Prince Edward Island), which is geopolitically a part of South Africa, and for which the fauna has been thoroughly investigated (Table 3, Gabriel et al. 2001, Hugo et al. 2006, Chown and Froneman 2008). Such a general situation of poor knowledge is typical for the Collembola in many parts of the world (e.g. Cicconardi et al. 2013), and will hamper efforts both to conserve this diversity (Cardoso et al. 2011) and to understand which components of it are non-indigenous and may be having impacts on the indigenous fauna (see discussion in Roques et al. 2009).

**Table 3.** Species recorded from the Prince Edward Islands, an island group geopolitically part of South Africa. Abbreviations used: E = endemic to Marion Island, S = sub-Antarctic distribution, I = introduced, D = dubious.

Current species name	Source	Status	Name in source and comments
**PODUROMORPHA**			
**Hypogastruridae**			
*Ceratophysella denticulata* (Bagnall, 1941)	Deharveng (1981)	I	Ceratophysella cf. denticulata (Bagnall, 1941)
*Hypogastrura viatica* (Tullberg, 1872)	Deharveng (1981)	D	Not found again since 1981, possible contamination (CJS pers. obs.)
**Neanuridae**			
*Friesea tilbrooki* Wise, 1970	Deharveng (1981)	S	*Friesea viennei* Deharveng, 1981 (syn Greenslade 1986)
**Tullbergiidae**			
*Tullbergia bisetosa* Börner, 1902	Deharveng (1981)	S	
**ENTOMOBRYOMORPHA**			
**Isotomidae**			
*Cryptopygus antarcticus travei* Deharveng, 1981	Deharveng (1981)	E	
*Cryptopygus dubius* Deharveng, 1981	Deharveng (1981)	S	
*Cryptopygus tricuspis* Enderlein, 1909	Deharveng (1981)	S	
*Folsomotoma marionensis* (Deharveng, 1981)	Deharveng (1981)	E	Isotoma (Sorensia) marionensis Deharveng, 1981
*Isotomurus maculatus* Müller, 1876	Deharveng (1981)	I	Isotomurus cf. palustris, confirmed as *Isotomurus maculatus* by Greenslade (2010)
*Mucrosomia caeca* (Wahlgren, 1906)	Deharveng (1981)	S	*Cryptopygus caecus* Wahlgren, 1906 (new comb. after Potapov 2001)
*Parisotoma notabilis* (Schäffer, 1896)	Deharveng (1981)	I	Isotoma (Parisotoma) notabilis
**Tomoceridae**			
*Pogonognathellus flavescens* (Tullberg, 1871)	Gabriel et al. (2001)	I	
**NEELIPLEONA**			
**Neelidae**			
*Megalothorax minimus* Willem, 1900	Deharveng (1981)	I	Megalothorax cf. minimus Willem, 1900, identification confirmed by C. Schneider (pers. comm.)
**SYMPHYPLEONA**			
**Katiannidae**			
*Sminthurinus granulosus* Enderlein, 1909	Deharveng (1981)	S	Sminthurinus cf. granulosus Enderlein, 1909 in Deharveng (1981)
*Sminthurinus tuberculatus* Delamare Deboutteville & Massoud, 1963	Gabriel et al. (2001)	S	Sminthurinus cf. kerguelensis Salmon, 1964 in Deharveng (1981)
*Katianna* sp.	Chown and Froneman (2008)	E	

With the caveat in mind of undersampling, both in many parts of Africa and country-wide, it is worth considering what the current information on species in the country suggests. It appears that endemicity is likely to be high (currently 65%). This value is similar to that found for other invertebrate groups and plants in South Africa, with an extraordinary high number of endemic species found in the south-western Cape (see Colville et al. 2002, Goldblatt and Manning 2002, Herbert and Kilburn 2004, Rebelo et al. 2006, Pryke and Samways 2010). Endemicity is expected to increase with local sampling, but will likely decline if sampling is undertaken in neighbouring countries where information on the group is similarly low (e.g. Namibia, see Thibaud and Massoud 1988). Currently, sampling in the southern part of Africa mostly concerns sites within South Africa, generating a rapid increase in species richness and endemicity, as many additional endemic species have been obtained from samples as little as a few kilometres from already well sampled areas (Janion-Scheepers, Bedos and Deharveng unpublished results).

Currently, six genera are thought to be endemic to South Africa: *Najtafrica* Barra, 2002 (one species, Pseudachorutinae), *Probrachystomellides* Weiner & Najt, 1991 (one species, Brachystomellidae), *Capbrya* Barra, 1999 (two species, Entomobryidae), *Lepidokrugeria* Coates, 1969 (one species, Lepidocyrtinae), *Neophorella* Womersley, 1934 (one species, Tomoceridae) and *Tritosminthurus* Snider, 1988 (one species, Bourletiellidae). *Neophorella dubia* was described from a single specimen by Womersley (1934) and is the only endemic species of the family Tomoceridae to occur in South Africa. Paclt (1959) mentioned that besides the single holotype specimen, this species was not found again and he synonymised it with the Paronellidae *Dicranocentruga nigromaculata* (Schött, 1903). Ireson and Greenslade (1990) re-examined the type specimen and re-assigned the species to Tomoceridae, stressing however its similarity with Isotomidae (Skaife 1954). In spite of intensive sampling in its type locality of Table Mountain (Janion-Scheepers, Bedos and Deharveng unpublished results), the species was not retrieved in any of our samples, and is considered here as a *species inquirenda*.

The current information also suggests that approximately 20% of the Collembola species found in South Africa may have been introduced by humans to the region and should therefore be considered alien (see Pyšek et al. 2004 for terminology). Understanding what the proportion of introduced species in the fauna actually is will depend on additional comprehensive sampling, and on further consideration of species currently though to be alien. Thus, several species resembling well-known European taxa had previously been mistakenly assigned to these taxa. For example, *Seira squamoornata*, which was originally described from the Ukraine, was thought to be a common polymorphic species in South Africa after Paclt (1959). However, Yosii (1959) did not even include this species in his list, while Coates (1968b) found that specimens labelled as one species (*Seira squamoornata*) by Paclt (1959), could actually be identified as several endemic species described by Yosii (1959) or Coates (1968b), and concluded that this European species does not occur in South Africa. Indeed, to date 25 indigenous species of *Seira* have been described from South Africa (Yosii 1959, Coates 1968b), and the richness of the genus is likely much larger.

Nonetheless, that several alien species are present, especially of European origin, is not surprising given the close historical links between South Africa and Europe (Giliomee and Mbenga 2007). Most of the invasive species were collected in disturbed environments, in gardens or close to human settlements (Supplementary Material Suppl. material 1) bearing out findings for a range of other groups that disturbance may favour alien species establishment (Chytrý et al. 2005, MacDougall and Turkington 2005, Richardson and Pyšek 2006). Perhaps the best known of the alien species is *Sminthurus viridis*, also known as the Lucerne flea (Wallace 1964, Wallace and Walters 1974), which received considerable attention in South Africa during the late 1960s due to its pest status. It is thought to have arrived from Australia as eggs in soil through the importation of clover seed (Walters 1968, Wallace and Walters 1974). It was first collected in 1951 near Somerset West and by 1959 over 50 000 hectares of Lucerne were infested (Wallace and Walters 1974). The problem now appears largely to have been resolved, although the species is still listed as a pest of Lucerne (Annecke and Moran 1982).

In conclusion, based on published knowledge only, the Collembola species richness of South Africa is high compared with other African countries (Thibaud 2013), but low compared with non-African countries (Deharveng 2007) and with the richness of other invertebrate groups in the South African region (Scholtz and Chown 1995). This is likely due to undersampling, as recent discoveries (e.g. Janion et al. 2011b, Potapov et al. 2011, Janion et al. 2012, 2013) have indicated. Owing to a recent, large and comprehensive ecological and systematic study, accompanied by DNA Barcoding (Porco et al. 2012) largely focused on the country’s Western Cape Province (Bengtsson et al. 2010, Janion et al. 2011a, Liu et al. 2012), a substantial increase in the number of species is expected. With 67 species recognised for the Western Cape from the recorded literature, the richness estimates indicating at least 6–7 times that number being present, and based on experience in other undersampled countries such as Thailand (Bedos 1994), we expect that species richness for the country will exceed 1000. Improvement of systematic knowledge through studies such as these, and improvements in ecological understanding of the impacts of both landscape change and invasive species on the springtail fauna (e.g. Gabriel et al. 2001, Liu et al. 2012), will help South Africa meet its commitments to biodiversity conservation especially as set out in the 2020 Aichi Biodiversity Targets.

## Supplementary materials

Supplementary material 1 Table S1 Data type: occurrence Explanation note: Collection details of Collembola recorded from continental South Africa. This dataset is made available under the Open Database License (http://opendatacommons.org/licenses/odbl/1.0/). The Open Database License (ODbL) is a license agreement intended to allow users to freely share, modify, and use this Dataset while maintaining this same freedom for others, provided that the original source and author(s) are credited.Charlene Janion-Scheepers, Louis Deharveng, Anne Bedos, Steven L. Chown

## References

[B1] Abrantes EA Bellini BC Bernardo AN Fernandes LH Mendonça MC Oliveira EP Queiroz GC Sautter KD Silveira TC Zeppelini D (2010) Synthesis of Brazilian Collembola: an update to the species list. Zootaxa 2388: 1–22. http://www.mapress.com/zootaxa/2010/f/z02388p022f.pdf

[B2] Abrantes EA Bellini BC Bernardo AN Fernandes LH Mendonça MC Oliveira EP Queiroz GC Sautter KD Silveira TC Zeppelini D (2012) Errata Corrigenda and update for the ‘Synthesis of Brazilian Collembola: an update to the species list.’ ABRANTES et al. (2010), Zootaxa, 2388: 1–22. Zootaxa 3168: 1–21. http://www.mapress.com/zootaxa/2012/f/z03168p021f.pdf

[B3] Annecke DP Moran VC (1982) Insects and mites of cultivated plants in South Africa. Butterworths, Durban.

[B4] Bacher S (2012) Still not enough taxonomists: reply to Joppa et al. Trends in Ecology and Evolution 27: 65–66. doi: 10.1016/j.tree.2011.11.00310.1016/j.tree.2011.11.003 22138045 10.1016/j.tree.2011.11.003

[B5] Barra JA (1994) Nouveaux Collemboles Poduromorphes de la Province du Natal (Rép. Sud Africaine) (Insecta: Collembola). Journal of African Zoology 108: 181–189.

[B6] Barra JA (1995) Nouveaux Collemboles Poduromorphes des sables littoraux (partie terrestre) de la Province du Natal (Rép. Sud Africaine) (Insecta: Collembola). Journal of African Zoology 109: 125–139.

[B7] Barra JA (1997) Nouveaux Collemboles Entomobryomorphes des sables littoraux (partie terrestre) de la Province du Natal (Rép. Sud Africaine) (Insecta : Collembola). Journal of African Zoology 111: 465–480.

[B8] Barra JA (1999) Un nouveau genre *Capbrya* avec deux nouvelles espèces de la Province du Cap (Rep. Sud Africaine) (Collembola: Entomobryidae). Bulletin de l’Institut Royal des Sciences Naturelles de Belgique, Entomologie 69: 19–24.

[B9] Barra JA (2001) *Proisotoma davidi* sp. n. from Cape Province (South Africa) (Collembola). Deutsche Entomologische Zeitschrift 48: 23–26. doi: 10.1002/mmnd.4800480103

[B10] Barra JA (2002) Un nouveau genre de Pseudachorutinae (Collembola) des sables littoraux de la Province du Natal (République Sud africaine). Zoosystema 24: 177–180.

[B11] Barra JA Weiner WM (2009) A new species of *Delamarephorura* Weiner & Najt, 1999 (Collembola,Tullbergiidae) from Cape Province (South Africa). Acta Zoologica Cracoviensia 52B: 57–60. doi: 10.3409/azc.52b_1-2.57-60

[B12] Bellinger PF Christiansen KA Janssens F (1996–2014) Checklist of the Collembola of the World. http://www.collembola.org [accessed 10 November 2014]

[B13] Bedos A (1994) Les Collemboles édaphiques du massif du Doi Inthanon (Thailande): biodiversité et écologie en forêt tropicale. PhD thesis, Toulouse III University, Toulouse, France.

[B14] Bengtsson J Janion C Chown SL Leinaas HP (2011) Variation in decomposition rates in the fynbos biome, South Africa: the role of plant species and plant stoichiometry. Oecologia 165: 225–235. doi: 10.1007/s00442-010-1753-7 20827492 10.1007/s00442-010-1753-7 PMC3015188

[B15] Bengtsson J Janion C Chown SL Leinaas HP (2012) Litter decomposition in fynbos vegetation, South Africa. Soil Biology and Biochemistry 47: 100–105. doi: 10.1016/j.soilbio.2011.11.023

[B16] Berlese JE (1905) Apparecchio per racogliere press ed in gran numiro piccoli artropodi. Redia 2: 85–89.

[B17] Betsch JM (1980) Eléments pour une monographie des Collemboles Symphypléones (Hexapodes, Apterygotes). Mémoires du Muséum national d'Histoire naturelle 116: 1–227.

[B18] Biggs R Simons H Bakkenes M Scholes RJ Eickhout B van Vuuren D Alkemade R (2008) Scenarios of biodiversity loss in southern Africa in the 21st century. Global Environmental Change 18: 296–309. doi: 10.1016/j.gloenvcha.2008.02.001

[B19] Börner C (1908) Collembolen aus Südafrika, nebst einer Studie über die I. Maxille der Collembolen. In: Schultze L (Ed.) Forschungsreise im westlichen und zentralen Südafrika. Denkschriften der Medicinisch-naturwissenschaftlichen Gesellschaft zu Jena 13: 53–68.

[B20] Börner C (1913) Neue Cyphoderinen. Zoologisher Anzeiger 41: 274–284.

[B21] Bretfeld G (1999) Symphypleona. In: Dunger W (Ed.) Synopses on Palaearctic Collembola, Vol. 2. Abhandlungen und Berichte des Naturkundemuseums Görlitz 71: 1–318.

[B22] Brown JM (1926) Some African Apterygota. Annals and Magazine of Natural History 9: 34–44.

[B23] Butchart SHM Walpole M Collen B van Strien A Scharlemann JPW Almond REA Baillie JEM Bomhard B Brown C Bruno J Carpenter KE Carr GM Chanson J Chenery AM Csirke J Davidson NC Dentener F Foster M Galli A Galloway JN Genovesi P Gregory RD Hockings M Kapos V Lamarque J-F Leverington F Loh J McGeoch MA McRae L Minasyan A Morcillo MH Oldfield TEE Pauly D Quader S Revenga C Sauer JR Skolnik B Spear D Stanwell-Smith D Stuart SN Symes A Tierney M Tyrrell TD Vié J-C Watson R (2010) Global biodiversity: indicators of recent declines. Science 328: 1164–1168. doi: 10.1126/science.1187512 20430971 10.1126/science.1187512

[B24] Cardoso P Erwin TL Borges PAV New TR (2011) The seven impediments in invertebrate conservation and how to overcome them. Biological Conservation 14: 2647–2655. doi: 10.1016/j.biocon.2011.07.024

[B25] Chen JX Christiansen K (1997) Subgenus *Coecobrya* of the genus *Sinella* (Collembola: Entomobryidae) with special reference to the species of China. Annals of the Entomological Society of America 90: 1–19. doi: 10.1093/aesa/90.1.1

[B26] Christiansen K Bellinger P (1998) The Collembola of North America north of the Rio Grande, second edition Grinnell College, Grinnell, IA, 1520 pp.

[B27] Chown SL (2010) Temporal biodiversity change in transformed landscapes: a southern African perspective. Philosophical Transactions Royal Society B 365: 3729–3742. doi: 10.1098/rstb.2010.0274 10.1098/rstb.2010.0274 PMC2982005 20980320

[B28] Chown SL Froneman PW (2008) The Prince Edward Islands. Land-sea Interactions in a changing climate. African Sun Media, Stellenbosch.

[B29] Chytrý M Pyšek P Tichý L Knollová I Danihelka J (2005) Invasions by alien plants in the Czech Republic: a quantitative assessment across habitats. Preslia 77: 339–354. http://www.preslia.cz/P054CChy.pdf

[B30] Cicconardi F Fanciulli PP Emerson BC (2013) Collembola, the biological species concept and the underestimation of global species richness. Molecular Ecology 22: 5382–5396. doi: 10.1111/mec.12472 24112308 10.1111/mec.12472

[B31] Coates TJ (1968a) The Collembola from South Africa – I: The Genus *Neanura*. Journal of the Entomological Society of Southern Africa 31: 185–195. http://content.ajarchive.org/cdm4/document.php?CISOROOT=/00128789&CISOPTR=3122&REC=1

[B32] Coates TJ (1968b) The Collembola of South Africa – 2: The Genus *Seira*. Journal of the Entomological Society of Southern Africa 31: 435–462. http://content.ajarchive.org/cdm4/document.php?CISOROOT=/00128789&CISOPTR=3122&REC=1

[B33] Coates TJ (1969) The Collembola of South Africa – 3: The Genus *Lepidokrugeria*. Journal of the Entomological Society of Southern Africa 32: 87–89. http://content.ajarchive.org/cdm4/document.php?CISOROOT=/00128789&CISOPTR=4010&REC=2/

[B34] Coates TJ (1970) Check-list of the Collembola of South African Parks. Koedoe 13: 181–184. doi: 10.4102/koedoe.v13i1.740

[B35] Colville J Picker MD Cowling RM (2002) Species turnover of monkey beetles (Scarabaeidae: Hopliini) along environmental and disturbance gradients in the Namaqualand region of the succulent Karoo, South Africa. Biodiversity and Conservation 11: 243–264. doi: 10.1023/A:1014520226989

[B36] Colwell RK (2009) EstimateS – statistical estimation of species richness and shared species from samples. Version 8.2.0. http://viceroy.eeb.uconn.edu/EstimateSPages/EstimateS.flx

[B37] Costello MJ May RM Stork NE (2013) Can we name Earth’s species before they go extinct? Science 339: 413–416. doi: 10.1126/science.1230318 23349283 10.1126/science.1230318

[B38] Culik MP Zeppelini D (2003) Diversity and distribution of Collembola (Arthropoda: Hexapoda) of Brazil. Biodiversity Conservation 12: 1119–1143. doi: 10.1023/A:1023069912619

[B39] Davis ALV (1997) Climatic and biogeographical associations of southern African dung beetles (Coleoptera: Scarabaeidae *s. str.*). African Journal of Ecology 35: 10–38. doi: 10.1111/j.1365-2028.1997.051-89051.x

[B40] Deharveng L (1981) Collemboles des îles subantarctiques de l’océan Indien. Comité National Française des Recherches Antarctiques 48: 33–108.

[B41] Deharveng L (2004) Recent advances in Collembola systematics. Pedobiologia 48: 415–433. doi: 10.1016/j.pedobi.2004.08.001

[B42] Deharveng L (2007) Collembola. Fauna Europaea version 1.3. http://www.faunaeur.org [accessed 02 April 2014]

[B43] Deharveng L Dalens H Drugmand D Simon-Benito JC Da Gama MM Sousa P Gers C Bedos A (2000) Endemism mapping and biodiversity conservation in western Europe: an arthropod perspective. Belgian Journal of Entomology 2: 59–75. http://www.srbe-kbve.be/cm/sites/default/files/publications/BJE/Sommaire%20BJE%20papier/BJE-2000-vol2-%281-2%29.pdf

[B44] Deharveng L Lips J Rahmadi C (2011) Focus on guano. In: Bouchet P Le Guyader H Pascal O (Eds) The Natural History of Santo: Caves and soils. MNHN, Paris; IRD, Marseille; PNI, Paris. Patrimoines naturels 70: 300–305.

[B45] Dippenaar-Schoeman A (2014) The field guide to spiders of South African. Lapa Publishers, Pretoria.

[B46] Dippenaar-Schoeman AS González Reyes AX (2006) South African National Survey (SANSA): Solifugae (sun-spiders) of the national parks and reserves of South Africa (Arachnida, Solifugae). Koedoe 49: 29–38. doi: 10.4102/koedoe.v49i2.114

[B47] Dippenaar-Schoeman AS González Reyes AX Harvey M (2006) A check-list the Solifugae (sun-spiders) of South Africa (Arachnida, Solifugae). African Plant Protection 12: 70–92. http://content.ajarchive.org/cdm4/document.php?CISOROOT=/10233121&CISOPTR=276&REC=20

[B48] Dirzo R Raven PH (2003) Global state of biodiversity and loss. Annual Review of Environment and Resources 28: 137–167. doi 10.1146/annurev.energy.28.050302.105532

[B49] Erasmus BFN Kshatriya M Mansell MW Chown SL van Jaarsveld AS (2000) A modelling approach to antlion (Neuroptera: Myrmeleontidae) distribution patterns. African Entomology 8: 157–168. https://insects.tamu.edu/research/neuropterida/neur_bibliography/edoc12/erasmus2000ref9764s-8117.pdf

[B50] Erasmus BFN van Jaarsveld AS Chown SL Kshatriya M Wessels KJ (2002) Vulnerability of South African animals taxa to climate change. Global Change Biology 8: 679–69. doi: 10.1046/j.1365-2486.2002.00502.x

[B51] ESRI (Environmental Systems Resource Institute) (2011) ArcGIS Desktop Release 10.1. ESRI, Redlands, California.

[B52] Evans KL Rodrigues ASL Chown SL Gaston KJ (2006) Protected areas and regional avian species richness in South Africa. Biology Letters 2: 184–188. doi: 10.1098/rsbl.2005.0435 17148358 10.1098/rsbl.2005.0435 PMC1618914

[B53] Foord SH Dippenaar-Schoeman AS Haddad CH (2011) South African spider diversity: African perspectives on the conservation of a mega-diverse group. In: Grillo O (Ed.) Changing Diversity in Changing Environment. InTech, Croatia. doi: 10.5772/24775

[B54] Foord SH Dippenaar-Schoeman AS van der Merwe M (2002) A check list of the spider fauna of the Western Soutpansberg, South Africa (Arachnida: Araneae). Koedoe 45: 35–43. doi: 10.4102/koedoe.v45i2.25

[B55] Gabriel AGA Chown SL Barendse J Marshall DJ Mercer RD Pugh PJA Smith VR (2001) Biological invasions on Southern Ocean islands: the Collembola of Marion Island as a test of generalities. Ecography 24: 421–430. doi: 10.1111/j.1600-0587.2001.tb00477.x

[B56] Giliomee H Mbenga B (2010) New History of South Africa. Tafelberg Publishers, Cape Town http://www.tafelberg.com/Books/2652

[B57] Godfray HCJ (2002) Challenges for taxonomy. Nature 417: 17–19. doi: 10.1038/417017a 11986643 10.1038/417017a

[B58] Goldblatt P Manning JC (2002) Plant diversity of the Cape region of Southern Africa. Annals of the Missouri Botanical Garden 89: 281–302. doi: 10.2307/3298566, http://www.missouribotanicalgarden.org/Portals/0/staff/PDFs/goldblatt/Capeflorapdf1.pdf

[B59] Goto H (1953) A species of Collembola, *Sinella coeca* (Schött) (Entomobryidae), new to South Africa. Entomologist’s Monthly Magazine 89: 165–166.

[B60] Greenslade P (n.d.) The Aquatic Springtails (Insecta: Collembola) of South Africa. http://www.ru.ac.za/static/departments/zoo/Martin/acollembola.html [accessed 10 November 2014]

[B61] Greenslade P (1986) Additions to collembolan fauna of Heard Island. Records of the South Australian Museum 197: 91–96.

[B62] Greenslade P (1994) Collembola. In: Houston WWK (Ed.) Zoological catalogue of Australia. Volume 22. Protura, Collembola, Diplura. CSIRO, Melbourne, 19–138.

[B63] Greenslade P (2002) Assessing the risk of exotic Collembola invading subantarctic islands: prioritising quarantine management. Pedobiologia 46: 338–344. doi: 10.1078/0031-4056-00141

[B64] Greenslade P (2010) Collembola fauna of the South Shetland Islands revisited. Antarctic Science 22: 233–242.

[B65] Greenslade P Ireson J Skarzynski D (2014) Biology and key to the Australian species of *Hypogastrura* and *Ceratophysella* (Collembola: Hypogastruridae). Austral Entomology 53: 53–74. doi: 10.1111/aen.12048

[B66] Haddad CR Dippenaar-Schoeman AS (2006) Epigeic spiders (Araneae) in pistachio orchards in South Africa. African Plant Protection 12: 12–22. http://content.ajarchive.org/cdm4/document.php?CISOROOT=/10233121&CISOPTR=276&REC=20

[B67] Herbert D Kilburn D (2004) Field guide to the land snails and slugs of eastern South Africa. Natal Museum, Pietermaritzburg.

[B68] Hlavac P (2007) Revision of the subtribe Clavigerodina and an annotated catalogue of South African Clavigeritae (Coleoptera: Staphylinidae: Pselaphinae). African Invertebrates 48: 65–92.

[B69] Hogg ID Hebert PDN (2004) Biological identification of springtails (Hexapoda: Collembola) from the Canadian Arctic, using mitochondrial DNA barcodes. Canadian Journal of Zoology 82: 749–754. doi: 10.1139/z04-041

[B70] Hopkin S (1997) Biology of the Springtails. Insecta: Collembola. Oxford University Press, Oxford.

[B71] Hugo EA Chown SL McGeoch MA (2006) The microarthropods of sub-Antarctic Prince Edward Island: a quantitative assessment. Polar Biology 30: 109–119. doi: 10.1007/s00300-006-0166-x

[B72] Hugo-Coetzee EA Avenant NL (2011) The effect of fire on soil oribatid mites (Acari: Oribatida) in a South African grassland. Zoosymposia 6: 210–220. http://www.mapress.com/zoosymposia/content/2011/v6/f/v006p210-220f.pdf

[B73] Huntley B Barnard P (2012) Potential impacts of climatic change on southern African birds of fynbos and grassland biodiversity hotspots. Diversity and Distributions 18: 769–781. doi: 10.1111/j.1472-4642.2012.00890.x

[B74] Ireson JE Greenslade P (1990) *Lasofinius* gen. n. (Collembola: Tomoceridae) from Tasmania and a re-examination of *Neophorella dubia* Womersley (Tomoceridae). Journal of the Australian Entomological Society 29: 205–214. doi: 10.1111/j.1440-6055.1990.tb00350.x

[B75] Janion C Bedos A Bengtsson J Deharveng L Jansen van Vuuren B Leinaas HP Liu A Malmström A Porco D Chown SL (2011a) Springtail diversity in South Africa. South African Journal of Science 107: . doi: 10.4102/sajs.v107i11/12.582

[B76] Janion C Bedos A Deharveng L (2011b) The genus *Ectonura* Cassagnau, 1980 in South Africa (Collembola: Neanuridae: Neanurinae), with a key to South African Neanurinae. ZooKeys 136: 31–45. doi: 10.3897/zookeys.136.1744 22140347 10.3897/zookeys.136.1744 PMC3229287

[B77] Janion C D’Haese C Deharveng L (2012) A new species and first record of the genus *Triacanthella* Schaffer, 1897 (Collembola, Hypogastruridae) for Africa. ZooKeys 163: 57–68. doi: 10.3897/zookeys.163.2298 22303129 10.3897/zookeys.163.2298 PMC3253666

[B78] Janion C Deharveng L Weiner WM (2013) Synonymy of *Spicatella* Thibaud, 2002 with *Delamarephorura* Weiner and Najt, 1999, and description of two new species (Collembola: Tullbergiidae). Raffles Bulletin of Zoology 61: 657–663. http://lkcnhm.nus.edu.sg/nus/pdf/PUBLICATION/Raffles%20Bulletin%20of%20Zoology/Past%20Volumes/RBZ%2061(2)/61rbz657-663.pdf

[B79] Joppa LN Roberts DL Pimm SL (2011) The population ecology and social behaviour of taxonomists. Trends in Ecology and Evolution 26: 551–553. doi: 10.1016/j.tree.2011.07.010 21862170 10.1016/j.tree.2011.07.010

[B80] Jordana R (2012) Synopses on Palaearctic Collembola. Capbryinae and Entomobryini. Soil Organisms 84: 1–390.

[B81] Lawrence RF (1953) The biology of the cryptic fauna of forest. AA Balkema, Cape Town.

[B82] Liu A Janion C Chown SL (2012) Collembola diversity in the critically endangered Cape Flats Sand Fynbos and adjacent pine plantations. Pedobiologia 55: 203–209. doi: 10.1016/j.pedobi.2012.03.002

[B83] Longino JT Coddington J Colwell RK (2002) The ant fauna of a tropical rain forest: estimating species richness three different ways. Ecology 83: 689–702. doi: 10.1890/0012-9658(2002)083[0689:TAFOAT]2.0.CO;2

[B84] MacDougall AS Turkington R (2005) Are invasive species the drivers or passengers of change in degraded ecosystems? Ecology 86: 42–55. doi: 10.1890/04-0669

[B85] Magurran AE (2004) Measuring Biological Diversity. Blackwell Publishing, Oxford.

[B86] Massoud Z (1967) Monographie des Neanuridae, Collemboles Poduromorphes à pièces buccales modifiées. In: Biologie de l’Amérique Australe, vol.III C.N.R.S. (Ed.), Paris, 7–399.

[B87] Mitra SK (2002) Status of *Dicranocentruga* Wray, 1953 with the description of a new species (Collembola: Entomobryidae). Records of the Zoological Survey of India 100: 105–116.

[B88] Mucina L Rutherford MC (2006) The vegetation of South Africa, Lesotho and Swaziland. Strelitzia 19, South African National Biodiversity Institute, Pretoria http://www.sanbi.org/documents/strelitzia-19-vegetation-south-africa-lesotho-swaziland-2-cd-set

[B89] Nilsson-Örtman V Nilsson AN (2010) Using taxonomic revision data to estimate the global species richness and characteristics of undescribed species of diving beetles (Coleoptera: Dytiscidae). Biodiversity Informatics 7: 1–16. http://www.nilssonortman.com/images/pdfs/3631-5322-1-PB.pdf

[B90] Paclt J (1959) Collembola. South African Animal Life. Results of the Lund University Expedition in 1950–1951, Vol. VI Almqvist and Wiksells Boktryckeri AB, Uppsala, 24–78.

[B91] Paclt J (1964) Ein neuer *Prorastriopes* aus Zululand und zur Nomenklatur due südafrikianischen *Rastropes*-Art. Senckenbergiana Biologica 45: 661–663.

[B92] Paclt J (1965) *Cyphoderus trinervoidis* n.sp., ein neuer Termitophile aus Transvaal (Ins., Collembola). Senckenbergiana Biologica 46: 59–60.

[B93] Paclt J (1967) On South and Central African Collembola. Journal of the Entomological Society of South Africa 29: 135–47.

[B94] Parr CL Robertson HG Chown SL (2003) Apomyrminae and Aenictogitoninae: two new subfamilies of ant (Hymenoptera: Formicidae) for southern Africa. African Entomology 11: 128–129.

[B95] Petersen H Luxton M (1982) A comparative analysis of soil fauna populations and their role in decomposition processes. Oikos 39: 287–388. doi: 10.2307/3544689

[B96] Pimm SL Jenkins CN Joppa LN Roberts DL Russell GJ (2010) How many endangered species remain to be discovered in Brazil? Natureza and Conservação 8: 71–77. doi: 10.4322/natcon.00801011

[B97] Platnick NI (2000–2014) Merrett P Cameron HD (Eds) The World Spider Catalog, Version 14.5. American Museum of Natural History, NY http://research.amnh.org/iz/spiders/catalog

[B98] Porco D Bedos A Greenslade P Janion C Skarżyński D Stevens M Jansen van Vuuren B Deharveng L (2012) Challenging species delimitation in Collembola: cryptic diversity among common springtails unveiled by DNA barcoding. Invertebrate Systematics 26: 470–477. doi: 10.1071/IS12026

[B99] Potapov M (2001) Synopses on Palaearctic Collembola, Volume 3. Isotomidae. Abhandlungen und Berichte des Naturkundemuseums Görlitz, Band 73, Heft 2, 2001, 603.

[B100] Potapov M Gao Y Deharveng L (2013) Taxonomy of the *Cryptopygus* complex. I. *Pauropygus* – a new worldwide littoral genus (Collembola, Isotomidae). ZooKeys 304: 1–16. doi: 10.3897/zookeys.304.4083 23794906 10.3897/zookeys.304.4083 PMC3689120

[B101] Potapov M Janion C Deharveng L (2011) Two new species of *Parisotoma* (Collembola: Isotomidae) from the Western Cape, South Africa. Zootaxa 2771: 17–24. PMid:21547000, PMCid:3086760

[B102] Pryke JS Samways MJ (2010) Significant variables for the conservation of mountain invertebrates. Journal of Insect Conservation 13: 627–641. doi: 10.1007/s10841-009-9213-6

[B103] Pyšek P Richardson DM Rejmánek M Webster GL Williamson M Kirschner J (2004) Alien plants in checklists and floras: towards better communication between taxonomists and ecologists. Taxon 53: 131–143. doi: 10.2307/4135498

[B104] Rebelo AG Boucher C Helme N Mucina L Rutherford MC (2006) Fynbos Biome. In: Mucina L Rutherford MC (Eds) The vegetation of South Africa, Lesotho and Swaziland. Strelitzia 19, South African National Biodiversity Institute, Pretoria, 52–219.

[B105] Richardson DM Pyšek P (2006) Plant invasions: merging the concepts of species invasiveness and community invasibility. Progress in Physical Geography 30: 409–431. doi: 10.1191/0309133306pp490pr

[B106] Robertson HG (2000) Afrotropical ants (Hymenoptera: Formicidae): taxonomic progress and estimation of species richness. Journal of Hymenoptera Research 9: 71–84.

[B107] Robertson HG (2002) Revision of the ant genus *Streblognathus* (Hymenoptera: Formicidae: Ponerinae). Zootaxa 97: 1–16.

[B108] Roques A Rabitsch W Rasplus J-Y Lopez-Vaamonde C Nentwig W Kenis M (2009) Alien terrestrial invertebrates in Europe. In: Handbook of alien species in Europe. Springer, Heidelberg, 63–79. doi: 10.1007/978-1-4020-8280-1_5

[B109] Rougerie R Decaens T Deharveng L Porco D James SW Chang C-H Richard B Potapov M Suhardjono Y Hebert PDN (2009) DNA barcodes for soil animal taxonomy. Pesquisa Agropecuária Brasileira 44: 789–801. doi: 10.1590/S0100-204X2009000800002

[B110] Rouget M Richardson DM Cowling RM Lloyd JW Lombard AT (2003) Current patterns of habitat transformation and future threats to biodiversity in terrestrial ecosystems of the Cape Floristic Region, South Africa. Biological Conservation 112: 63–85. doi: 10.1016/S0006-3207(02)00395-6

[B111] Rousse P van Noort S (2013) Revision of the Afrotropical Lycorininae (Ichneumonidae, Hymenoptera) with description of a new species from South Africa. Zootaxa 3666: 252–266. doi: 10.11646/zootaxa.3666.2.8 10.11646/zootaxa.3666.2.8 26217848

[B112] Rusek J (1971) Zur Taxonomie der Tullbergia (Mesaphorura) krausbaueri (Börner) und ihrer verwandten (Collembola). Acta Entomologica Bohemoslovaca 68: 188–206.

[B113] Salmon JT (1964) An Index to the Collembola, Volume 1 Royal Society of New Zealand Bulletin No. 7, Wellington, 1–144.

[B114] Samper C (2004) Taxonomy and environmental policy. Philosophical Transactions of the Royal Society B 359: 721–728. doi: 10.1098/rstb.2004.1476 10.1098/rstb.2004.1476 PMC1693350 15253357

[B115] Schoeman CS Foord SH (2012) A checklist of ants (Hymenoptera: Formicidae) from the Marakele National Park, Limpopo, South Africa. Koedoe 54: . doi: 10.4102/koedoe.v54i1.1030

[B116] Scholtz CH Chown SL (1995) Insects in southern Africa: How many species are there? South African Journal of Science 91: 124–126.

[B117] Silvestri F (1918) Contribuzione alla conoscenza dei Termitidi e Termitofili dell’ Africa occidentale. II. Termitofili. Parte prima. Bollettino del Laboratorio di Zoologia General e Agraria, Portici 12: 287–346.

[B118] Skaife SH (1954) African Insect Life. Longmans Green, Cape Town.

[B119] Snider RJ (1988) *Tritosminthurus schuhi*, a new genus and species from Cape Province, South Africa (Collembola: Bourletiellidae). Entomological News 99: 260–266. http://biostor.org/cache/pdf/f0/a6/5b/f0a65bc6e8c19f64c0bec81e8ff44054.pdf

[B120] Stach J (1947) The Apterygotan fauna of Poland in relation to the world-fauna of this group of insects. Family Isotomidae, 488 pp.

[B121] Terauds A Chown SL Bergstrom DM (2011) Spatial scale and species identity influence the indigenous-alien diversity relationship in springtails. Ecology 92: 1436–1447. doi: 10.1890/10-2216.1 21870618 10.1890/10-2216.1

[B122] Thibaud JM (2008) Les collemboles des sables littoraux de Madagascar. Annales de la Société Entomologique de France 44: 503–519. doi: 10.1080/00379271.2008.10697586

[B123] Thibaud JM (2010) Les collemboles des sables littoraux de l’île de Mayotte. Essai de synthèse sur les collemboles des sables littoraux d’îles de l’Océan Indien (zone ouest). Revue Française d'Entomologie 32: 113–121.

[B124] Thibaud JM (2013) Essai sur l’état des connaissances de la diversité des Collemboles de l’Empire Africano–Malgache. Russian Entomological Journal 22: 233–248. http://zmmu.msu.ru/files/images/spec/Russ%20Ent%20J/ent22_4_233_248_Thibaud_new.pdf

[B125] Thibaud JM Massoud Z (1988) Recherche sur la faune interstitielle aérienne des sables fins: les Collemboles. II - Désert de Namibie. Annales de la Société Entomologique de France 24: 211–214.

[B126] Tullgren A (1918) Ein sehr einfacher Ausleseapparat fur terricole Tierformen. Zeitschrift Fur Angewante Entomologie 4: 149–150. http://zmmu.msu.ru/files/images/spec/Russ%20Ent%20J/ent22_4_233_248_Thibaud_new.pdf

[B127] van Noort S (2004–2014) Wasps, bees and ants of Africa and Madagascar. Iziko Museums of South Africa www.waspweb.org [accessed 02 April 2014]

[B128] van Straalen NM (1998) Evaluation of bioindicator systems derived from soil arthropod communities. Applied Soil Ecology 9: 429–437. doi: 10.1016/S0929-1393(98)00101-2

[B129] Wallace MMH (1964) Present and probable world distribution of *Sminthurus viridis* and prospects for its biological control. Pedobiologia 14: 238–243.

[B130] Wallace MMH Walters MC (1974) The introduction of *Bdellodes lapidaria* (Acari: Bdellidae) from Australia into South Africa for the biological control of *Sminthurus viridis* (Collembola). Australian Journal of Zoology 22: 505–517. doi: 10.1071/ZO9740505

[B131] Walters MC (1968) A study of *Sminthurus viridis* (L.) (Collembola) in the Western Cape Province. Entomology Memoirs 16: Department of Agriculture Technical Services. University of Stellenbosch, Stellenbosch, 99 pp.

[B132] Wardle DA Bardgett RD Klironomos JN Setälä H van der Putten WH Wall DH (2004) Ecological linkages between aboveground and belowground biota. Science 304: 1629–1633. http: 10.1126/science.1094875 15192218 10.1126/science.1094875

[B133] Weiner WM Najt J (1991) Collembola Poduromorpha of South Africa. Bonner zoologischer Beiträge 42: 369–387. http://alt.zfmk.de/BZB/1991/1991%20Weiner%20W.M.%20u.%20Najt%20J.%20p369.pdf

[B134] Weiner WM Najt J (1998) Collembola (Entognatha) from East Africa. European Journal of Entomology 95: 217–237.

[B135] Weiner WM Najt J (1999) New genus of Tullbergiinae (Collembola). Annales de la Société Entomologique de France 35: 183–187.

[B136] Womersley H (1931) Some Collembola of the family Sminthuridae from South Africa. Annals of the South African Museum 30: 137–156.

[B137] Womersley H (1934) On some Collembola-Arthropleona from South Africa and Southern Rhodesia. Annals of the South African Museum 30: 441–475.

[B138] Yosii R (1959) Collembolan fauna of the Cape Province, with special reference to the genus *Seira* Lubbock. Special Publication from the Seto Marine Biological Laboratory. Biological results from the Japanese Antarctic Research Expeditions 6: 3–24.

[B139] Yosii R (1977) Critical checklist of the Japanese species of Collembola. Contributions of the Biological Laboratory of Kyoto University 25: 141–170.

[B140] Zhang F Deharveng L Chen JX (2009) New species and rediagnosis of *Coecobrya* (Collembola: Entomobryidae), with a key to the species of the genus. Journal of Natural History 43: 2597–2615.

