# Supplementary material for: Updated list of Collembola species currently recorded from South Africa
Source: Zookeys. 2015 May 11;(503):55–88. doi: 10.3897/zookeys.503.8966 (PMC4440272; doi:10.3897/zookeys.503.8966)
Supplement: Supplementary material 1 — Table S1 [file zookeys-503-055-s001.docx]

#### Supplementary material

**Table S1**: Details of Collembola described or recorded from continental South Africa. Abbreviations used: South Africa (SA); Western Cape (WC); Eastern Cape (EC); Kwazulu-Natal (KZN); Gauteng (G); Limpopo (L); Free State (FS); Northern Cape (NC); Mpumalanga (MP); North West Province (NWP); Lesotho (Les); endemic (E); introduced (I); widespread, thus recorded outside of South Africa but not considered introduced (W); juvenile specimen (juv); male (♂); female (♀), questionable/dubious identification (D). ISEA - Institute of Systematics and Evolution of Animals, Polish Academy of Sciences, Krakow, Poland; ISNB- l' Institut Royal des Sciences Naturelle de Belgique; MNHN - Museum national d'Histoire naturelle, Paris, France; MSPU - Moscow State Pedagogical University, Russia; Museum Lund – Museum of Lund; NIC - National Insect Collection, Pretoria; RMCA - Royal Museum of Central Africa at Tervuren, Belgium / Musee royal d'Afrique centrale Tervuren, Belgique; SAMC - South African Museum, Cape Town; SAMA - South Australian Museum, Adelaide, Australia. Endemic genera are indicated by an asterisk (*), while status and comments are given in Table 2. For references see main text.

| **Current species name** | **Source** | **SA** | **Records** | **Collection** |
| --- | --- | --- | --- | --- |
| **PODUROMORPHA** |  |  |  |  |
| **Hypogastruridae** |  |  |  |  |
| *Acherontiella thibaudi* Barra, 1994 | Barra 1994 | KZN | Type locality: Sodwana Bay National Park, KwaZulu-Natal, 300 km N of Durban, foot of the dune at top of beach, humid sand at 20 cm depth, under low pioneer vegetation, J.-P. Rieb leg. | Holotype ♂ and one paratype ♀ in RMCA, paratype ♂ at MNHN, Paris, other paratypes with Barra |
| *Austrogastrura lobata* (Yosii, 1959) | Yosii 1959 | WC | Table Mountain, 11.iii.1958, 2 ♂ examined. |  |
| *Ceratophysella armata* (Nicolet, 1842) | Coates 1970 | EC | Addo Elephant National Park, on reeds and also in soil, Caesar's dam. |  |
| *Ceratophysella armata* (Nicolet, 1842) | Paclt 1959 | EC | Dordrecht district, from heap of decomposing mealie cobs (about 180 specimens), July and October 1957, R. van Pletzen leg., also small poplar grove from soil containing decomposing leaves. |  |
| *Ceratophysella armata* (Nicolet, 1842) | Paclt 1959 | FS | Fouriesburg, surface of rain pool, Jan or April 1957, about 1800 specimens, Bloemfontein, under few pine trees, from soil containing large amounts of organic material, April 1957 (1 specimen), Bloemfontein, garden, soil with large amounts of organic plant material, April 1957 (5 specimens) - all R. van Pletzen leg. |  |
| *Ceratophysella armata* (Nicolet, 1842) | Paclt 1959 | G | Johannesburg, 10.iii.1958, SA Institute for Medical Research leg., (140 specimens). |  |
| *Ceratophysella armata* (Nicolet, 1842) | Paclt 1967 | G | Louis Trichardt, surface of pools of standing water, 3.viii.1939 (20 specimens AcV63); Idem, Elim Hospital, surface of pools of standing water, 3.viii.1939, 70 specimens AcV64 (all T. Kelly leg), Krugersdorp District, Blyvooruitsig, damp garden soil, 29.ii.1956, 20 specimens AcV65; 7 specimens AcV88, D.B. Wells leg. | NIC |
| *Ceratophysella armata* (Nicolet, 1842) | Paclt 1959 | KZN | Drakensberg 8 miles ENE from Rhodes, on stony ericaceous heath, alt 8000 ft., 9.iii.1951 SSAE leg, loc. 223. | 16 specimens Museum Lund |
| *Ceratophysella armata* (Nicolet, 1842) | Womersley 1934 | KZN | Inchanga, Nov. 1917, K.H. Barnard leg. |  |
| *Ceratophysella armata* (Nicolet, 1842) | Paclt 1967 | NC | Upington, banks of Orange River, Dec 1937, 2 specimens, AcV49 and 3 specimens AcV52, Graaff-Reinet 24.ix.1939, 3 specimens AcV71 and 29.ix.1939 1 specimens AcV73, D.v.V. Webb leg. | NIC |
| *Ceratophysella armata* (Nicolet, 1842) | Womersley 1934 | NC | Langklip Siding, Gordonia C.P. Aug. 1925, H.K. Barnard leg. |  |
| *Ceratophysella armata* (Nicolet, 1842) | Paclt 1959 | WC | Cape Peninsula, Cape Point Nature Reserve, sieved from wet leaves in low bush, 10.xii.1950 Loc. 79, Wynberg Cave Ravine, sieved from wet vegetable debris, 2400 ft, 18.xii.1950, Hout Bay, Skoorsteenkop, sieved from vegetable debris in dense indigenous forest, 600 ft., 28.i.1951 loc. 161 (all SSAE leg.) | Thousands of specimens Museum Lund |
| *Ceratophysella armata* (Nicolet, 1842) | Paclt 1967 | WC | Mamre Road, Waterkloof Farm, July 1937, 3 specimens AcV30Gordons Bay, 9.viii.1937, 14 specimens AcV35, 10.viii.1937, 3 specimens AcV38, 11.viii.1937, AcV40 and 13.viii.1937, 18 specimens AcV43; Velddrift, damp soil along banks of Berg River, 20.iii.1956, 16 specimens AcV90, Muizenberg, Boyes Drive Waterfall, damp soil and moss, 15.ix.1956, 9 specimens AcV98, Hout Bay, Chapman’s Peak Drive, damp soil and moss, 22.iv.1956, 23 specimens AcV102, Table Mountain Drive Waterfall, damp soil and moss, 5.v.1956, 27 specimens AcV104, Idem, Table Mountain Drive, humus and pine needles, 5.v.1956, 7 specimens AcV105 (all material D.v.V. Webb leg.). | NIC |
| *Ceratophysella armata* (Nicolet, 1842) | Womersley 1934 | WC | Stellenbosch 24 .vii.1930, 12.viii.1930, H.W. leg., Kloofnek, 27.viii.1930 (H.W. leg), Stellenbosch C.P. 28.viii.1927 (A.J. Hesse). |  |
| *Ceratophysella armata* var. *trispina* Womersley, 1934 | Womersley 1934 | WC | Stellenbosch, 1927, A.J. Hesse leg. | Type in SAMC |
| *Ceratophysella denticulata* (Bagnall, 1941) (cf.) | Liu et al. 2012 | WC | Pine and Cape Flats Sand Fynbos litter, July/August 2010 |  |
| *Ceratophysella longispina* (Tullberg, 1876) | Womersley 1934 | KZN | Ichanga, Nov 1917, K.H. Barnard leg. |  |
| *Ceratophysella longispina* (Tullberg, 1876) | Womersley 1934 | NC | Langklip Siding, Gordonia, Aug 1925, K.H. Barnard leg. |  |
| *Hypogastrura manubrialis* (Tullberg, 1876) | Paclt 1967 | KZN | Durban, African Mushroom Industries Ltd., 15.viii.1939, 90 specimens. | NIC |
| *Hypogastrura manubrialis* (Tullberg, 1876) | Paclt 1959 | NC | Kimberley. |  |
| *Hypogastrura manubrialis* (Tullberg, 1876) | Womersley 1934 | NC | Kimberley, Feb 1915, Miss Wilman leg. |  |
| *Hypogastrura manubrialis* (Tullberg, 1876) | Womersley 1934 | WC | Elsenburg 24.vii.1930, Rondebosch, C.T., 29.vii.1930, Stellenbosch, 24.vii.1930 and 29.viii.1930. |  |
| *Hypogastrura manubrialis neglectus* (Börner, 1901) | Womersley 1934 | WC | Stellenbosch, 29.viii.1930 Womersley leg. |  |
| *Hypogastrura purpurescens* (Lubbock, 1868) | Paclt 1959 | WC | Cape Peninsula, Cape Point Nature Reserve, sieved from wet leaves in low bush, 10.xii.1950, SSAE leg, Loc. 79. | 3 specimens, Museum Lund |
| *Hypogastrura purpurescens* (Lubbock, 1868) | Paclt 1967 | WC | Paarl, fallen leaves, 29.vii.1937, 1 specimen AcV29, D.v.V. Webb leg. | NIC |
| *Hypogastrura purpurescens* (Lubbock, 1868) | Womersley 1934 | WC | Outskirts of Cape Town, Womersley leg, 24.viii.1930. |  |
| *Hypogastrura sahlbergi* (Reuter, 1895) | Paclt 1959 | WC | Franschhoek Bosreserve, Upper Berg river, at stream, alt 1500 ft., 1.xi.1950, SSAE leg, loc 21. | 1 specimen Museum Lund |
| *Hypogastrura sahlbergi rosea* (Reuter, 1895) | Womersley 1934 | WC | Near top of Lion's Head, Cape Town, on damp rocks, 3.viii.1930, Womersley leg. |  |
| *Hypogastrura viatica* (Tullberg, 1872) | Paclt 1959 | WC | Seapoint, Cape Town, Carpenter leg. |  |
| *Hypogastrura viatica* (Tullberg, 1872) | Womersley 1934 | WC | Sea Point, Cape Town, shore pools, Sept 1929 Prof. W. D'Arcy Thompson leg. |  |
| *Hypogastrura* sp. | Lawrence 1953 | ? | No information given. |  |
| *Mesogastrura libyca* (Caroli, 1914) | Paclt 1959 | WC | Cape Peninsula, Cape Point Nature Reserve, sieved from wet leaves in low bush, 10.xii.1950 Loc. 79 (64 specimens), Hout Bay, Skoorsteenkop, sieved from vegetable debris in dense indigenous forest, 600 ft., 28.1.1951 (74 specimens), loc. 161 all SSAE leg. | Museum Lund |
| *Schaefferia (Typhlogastrura)* sp. | Sharratt et al. 2000 | WC | Table Mountain Peninsula caves, bat guano. Probably *Triacanthella madiba* Janion, D’Haese and Deharveng, 2012 described from the same kind of cave habitat on Table Mountain. |  |
| *Triacanthella madiba* Janion, D’Haese & Deharveng, 2012 | Janion et al. 2012 | WC | Holotype ♀ and 17 paratypes (9 on slides and 8 in alcohol), South Africa: Western Cape, Cape Town, Table Mountain National Park, 10 March 2009, bat guano in Wynberg cave, extracted on Berlese-Tullgren funnel, (SAF-125, Louis Deharveng & Anne Bedos leg). | Holotype on slide and 9 paratypes (5 on slides and 4 in alcohol) in SAMC, 8 paratypes in MNHN, (4 on slides and 4 in alcohol). |
| *Willemia trilobata* Barra, 1995 | Barra 1995 | KZN | Type locality: Sodwana Bay, foot of the dune at top of beach, humid sand at 20 cm depth, under low pioneer vegetation, 03.ii.1992, J.-P. Rieb leg. | Holotype ♂ and ♀ paratype, RMCA |
| *Xenylla capensis* Weiner and Najt, 1991 | Weiner & Najt 1991 | WC | Type locality: Saasveld, near George, subtropical indigenous forest, pitfalls, 17.xii.1986-17.i.1987, V. Nicolai leg. | Holotype ♀, paratypes 2 ♂, 3 ♀, 6 juv, (ISEA), paratypes 2 ♀, 1 ♂, 2 juv (MNHN) |
| *Xenylla maritima* Tullberg, 1869 | Paclt 1959 | EC | Dordrecht district, from heap of decomposing mealie cobs (about 180 specimens), July and October 1957, R. van Pletzen leg., also small poplar grove from soil containing decomposing leaves, a population of about 2500 specimens |  |
| *Xenylla maritima* Tullberg, 1869 | Paclt 1959 | KZN | Drakensberg, Champagne Castle, Feb 1946, R.F. Lawrence leg, 1 specimen. |  |
| *Xenylla maritima* Tullberg, 1869 | Paclt 1967 | NWP | Marico District, fallen leaves, 13.i.1930, one specimen AcV3, J.C. Faure leg. | NIC |
| *Xenylla maritima* Tullberg, 1869 | Paclt 1959 | WC | Cape Peninsula, Cape Point Nature Reserve, sieved from wet leaves in low bush, 10.xii.1950, loc. 79 (45 specimens), Table Mountain, Wynberg Cave Ravine, sieved from wet debris, 2400ft, 18.xii.1950 loc 84 (5 specimens), Hout Bay, Skoorsteenkop, sieved from vegetable debris in dense indigenous forest, 600ft, 28.i.1951, loc.161 (18 specimens), all SSAE leg., (Cape Town, on pods June 1915 K.H. Barnard leg. from Womersley slide). | Museum Lund |
| *Xenylla maritima* Tullberg, 1869 | Paclt 1967 | WC | Mamre Road, Waterkloof Farm, 3.vii 1937, 5 specimens AcV28 and July 1937, 46 specimens AcV30; Velddrift, damp soil along banks of Berg River, 20.iii.1956, 1 specimen AcV90; Van Rhynsdorp Knersvlakte, 3 miles from Van Rhyns Pass, 22.iii.1956, 2 specimens AcV93; Kalk Bay, Boyes Drive, damp soil, 22.iv.1956, 1 specimen AvC93, Rosebank, Entomology Research Station, damp soil and moss, 24.ix.1956, 6 specimens AcV103, Table Mountain Drive, humus and pine needles, 5.v.1956, 1 specimen AcV105, all material D.v.V. Webb leg. | NIC |
| *Xenylla maritima* Tullberg, 1869 | Womersley 1934 | WC | Stellenbosch – 12.xiii.1930, Fish Hoek – 23.viii.1930, Hout Bay - Aug 1930 (Womersley leg.) |  |
| *Xenylla rhodesiensis* Womersley, 1929 | Coates 1970 | MP | Kruger National Park, under reeds in vlei, Klopperfontein No. 151. |  |
| *Xenylla schillei* Börner, 1903 | Paclt 1959 | Les | Qachas Nek, at small stony stream surrounded by grassy meadow, alt 6500 ft., 7.iii.1951, SSAE leg, loc. 213 | 1 specimen Museum Lund |
| *Xenylla yucatana* Mills, 1938 | Barra 1995 | KZN | Sodwana Bay dense shrub forest, sand thin humus layer, 02.ii.1992, J-P Rieb leg. | RMCA |
| *Xenylla* sp. | Lawrence 1953 | ? | No information given. |  |
| **Brachystomellidae** |  |  |  |  |
| *Brachystomella africana* Yosii, 1959 | Yosii 1959 | WC | Kloofnek, 4.xii.1957, 12 specimens. |  |
| *Brachystomella coatesi* Weiner and Najt, 1991 | Weiner & Najt 1991 | WC | Type locality: Saasveld, near George, subtropical indigenous forest, pitfall traps, 17.xii.1986-17.i.1987, V. Nicolai leg | Holotype ♀, paratypes 1 ♂ juv, 10 ♀, 6 juv (ISEA), paratypes 7 ♀, 4 juv, (MNHN) |
| *Brachystomella georgensis* Weiner and Najt, 1991 | Weiner & Najt 1991 | WC | Type locality: Saasveld, near George, subtropical indigenous forest, pitfall traps, 17.xii.1986-17.i.1987, V. Nicolai leg | Holotype ♀, paratypes 2 ♀, 2 juv (ISEA), paratypes 1 ♀, 2 juv, (MNHN) |
| *Brachystomella parvula* (Schäffer, 1896) | Paclt 1959 | EC | Dordrecht district, from heap of decomposing mealie cobs, July and October 1957, 20 specimens, R. van Pletzen leg. |  |
| *Brachystomella parvula* (Schäffer, 1896) | Paclt 1959 | FS | Fouriesburg, surface of rain pool, Jan or April 1957, about 270 specimens, Bloemfontein, under few pine trees, from soil containing large amounts of organic material, April 1957, 1 specimen, Bloemfontein, garden, soil with large amounts of organic plant material, 11 specimens, R. van Pletzen leg. |  |
| *Brachystomella parvula* (Schäffer, 1896) | Paclt 1959 | KZN | Pietermaritzburg, from dry leaves in garden, Sept 1951, 12 specimens, Champagne Castle, Drakensberg, Feb 1946, 6 specimens, R.F. Lawrence leg. |  |
| *Brachystomella parvula* (Schäffer, 1896) | Coates 1970 | MP | Kruger National Park, under rotting leaves of *Bougainvillea* sp. and under *Portulacaria afra*, Shingwidzi Rest Camp; under *Hyphaene crinita*, Shingwidzi River. |  |
| *Brachystomella parvula* (Schäffer, 1896) | Paclt 1959 | WC | Cape Peninsula, Cape Point Nature Reserve, sieved from wet leaves in low bush, 10.12.1950 Loc. 79 (61 specimens), Hout Bay, Skoorsteenkop, sieved from vegetable debris in dense indigenous forest, 600 ft., 28.1.1951 loc. 161 (14 specimens) (all SSAE leg.). | All specimens Museum Lund |
| *Brachystomella parvula* (Schäffer, 1896) | Paclt 1967 | WC | Van Rhynsdorp Knersvlakte, 3 miles from Van Rhyns Pass, 22.iii.1956, 75 specimens AcV93; Muisenberg Boyes Drive Waterfall, damp soil and moss, 15.iv.1956, 1 specimens AcV98; Kalk Bay, Boyes Drive, damp soil, 22.iv.1956, 1 specimen AvC100, Simons Town, Red Hill Drive, damp soil and moss, 22.iv.1956, 4 specimens AcV101, Table Mountain Drive waterfall, damp soil and moss, 5.v.1956, 12 specimens AcV104, Ibidem, Table Mountain Drive, humus and pine needles, 5.v.1956, 7 specimens AcV105, all material D.v.V. Webb leg. | NIC |
| *Brachystomella parvula* (Schäffer, 1896) | Womersley 1934 | WC | Burghersdorp, Jan. 1913 Robertson leg, Cape Town, 9 May 1916 (K.H. Barnard leg), Elsenberg, 24 July 1920 (Womersley leg), Rondebosch C.P. July 1930 (Womersley leg), Kloof Nek, 3.viii.1930 (Womersley leg), Stellenbosch, 19 Aug. 1920 (Womersley leg) |  |
| *Brachystomella* sp. | Lawrence 1953 | ? | No information given. |  |
| *Brachystomella* sp. | Liu et al. 2012 | WC | Pine and Cape Flats Sand Fynbos litter, Tokai, July/August 2010 |  |
| *Probrachystomellides nicolaii* Weiner and Najt, 1991* | Weiner & Najt 1991 | WC | Type locality: Saasveld, near George, subtropical indigenous forest, pitfall traps, 17.xii.1986-17.i.1987, V. Nicolai leg. | Holotype ♀, paratypes 9 ♀, 5 ♂, 11 juv (ISEA), paratypes 11 ♀, 3 ♂, 8 juv, (MNHN) |
| *Setanodosa capitata* (Womersley, 1934) | Womersley 1934 | WC | Stellenbosch, 12.viii.1930 (Womersley leg), Cape Town June 1915 (K.H. Barnard leg). | Co-types in SAMC |
| *Setanodosa* sp. | Liu et al. 2012 | WC | Pine and Cape Flats Sand Fynbos litter, Tokai, July/August 2010 |  |
| **Neanuridae** |  |  |  |  |
| *Achorutes* sp. | Lawrence 1953 | ? | No information given. Invalid genus name. |  |
| *Aethiopella capensis* (Womersley, 1934) | Womersley 1934 | KZN | Inchanga, Nov. 1917, K.H.B. leg. |  |
| *Aethiopella capensis* (Womersley, 1934) | Paclt 1959 | WC | Bainskloof, 10 miles east of Wellington, at stony stream, mountain slope, 1.vii.1951, SSAE leg, - Ibidem, under stones on sandy ground, alt 6800 ft., 1.vii. 1951, SSAE leg, loc. 346. | 2 specimens Museum Lund, Ibidem 2 specimens |
| *Aethiopella capensis* (Womersley, 1934) | Womersley 1934 | WC | Slopes of Table Mountain, Cape Town, 5.viii.1919, K.H.B. leg, 29.viii.1930, Womersley leg. |  |
| *Aethiopella flavoantennata* (Philiptschenko, 1926) (cf.) | Liu et al. 2012 | WC | Pine litter, Tokai, July/August 2010 |  |
| *Aethiopella handschini* (Denis, 1924) | Paclt 1959 | Les | Mount Morosi, 15 miles NE of Quthing, under stone in wet ravine, alt 6600 ft., 18.3.1951, SSAE leg, loc 241. | 4 specimens Museum Lund |
| *Aethiopella handschini* (Denis, 1924) | Paclt 1959 | WC | Hout Bay, Skoorsteenkop, sieved from vegetable debris in dense indigenous forest, 600 ft., 28.i.1951, loc. 161. | 21 specimens Museum Lund (young specimens only) |
| *Anurida maritima* (Guérin-Méneville, 1836) | Lawrence 1953 | ? | No information given. |  |
| *Anurida maritima* (Guérin-Méneville, 1836) | Womersley 1934 | KZN | Durban, Jan 1913 (K.H. Barnard leg). |  |
| *Anurida maritima* (Guérin-Méneville, 1836) | Paclt 1959 | WC | Strandfontein, False Bay, Nov. 1930, K.H. Barnard leg |  |
| *Anurida maritima* (Guérin-Méneville, 1836) | Womersley 1934 | WC | Saldannha Bay 5.ix.1912, Cape Peninsula 1914, Melkbos Strand 28.x.1927, Kleinmond, Feb 1927, Hout Bay, 11.ii.1914 (all K.H. Barnard leg), Sea Point 31.vii.1930, Muizenberg 26.vii.1930 (Womersley leg). |  |
| *Anurida maritima* (Guérin-Méneville, 1836) | Yosii 1959 | WC | Seapoint, 30 specimens, 11.iii.1958. | Cosmopolitan distribution (Fjellberg 1998) |
| *Ectonura barrai* Janion, Bedos & Deharveng, 2011 | Janion et al. 2011 | WC | Type locality: Grootvadersbosch Nature Reserve, Heidelberg, 24.viii.2010, Southern Afrotemperate Forest vegetation, in litter, extraction on Berlese funnel, C. Janion leg (RSA10_GVB009, 33°59.167’S, 20°48.639’E). | Holotype and 4 paratypes (1 ♂ and 1 juv on slides, 2 in alcohol) in SAMC; 5 paratypes (1 ♂ and 1 juv on slides, 3 in alcohol) in MNHN |
| *Ectonura coatesi* Barra, 1994 | Barra 1994 | KZN | Sodwana Bay National Park, litter on dunes, 02.ii.1992, J.-P. Rieb leg. | Holotype ♂ and paratype ♀, RMCA, 2 ♀ paratypes with author |
| *Ectonura monochaeta* Janion, Bedos & Deharveng, 2011 | Janion et al. 2011 | WC | Type locality: Cape Town, Table Mountain National Park, 10. iii.2009, native forest, sieving of liter and extraction on Berlese funnel, l. Deharveng & A. Bedos leg, SAF141. | Holotype and 6 paratypes on slide (3 ♂, 3 ♀), 25 in alcohol in SAMC; 7 paratypes (3 ♂, 3 ♀ and 1 juv on slides), 25 in alcohol in MNHN |
| *Ectonura natalensis* (Womersley, 1934) | Paclt 1959 | EC | Dordrecht district, small poplar grove from soil containing decomposing leaves (about 18 specimens), October 1957, R. van Pletzen leg. |  |
| *Ectonura natalensis* (Womersley, 1934) | Paclt1959 | KZN | Pietermaritzburg, from dry leaves in garden, Sept 1951, 5 specimens, Champagne Castle, Drakensberg, Feb 1946, 1 specimens, R.F. Lawrence leg. |  |
| *Ectonura natalensis* (Womersley, 1934) | Womersley 1934 | KZN | Inchanga, K.H. Barnard, Nov. 1917 | Co-types in SAMC |
| *Ectonura natalensis* (Womersley, 1934) | Paclt 1959 | WC | Hout Bay, Skoorsteenkop, sieved from vegetable debris in dense indigenous forest, 600 ft., 28.i.1951 loc. 161 | 1 specimen Museum Lund |
| *Ectonura oribiensis* (Coates, 1968) | Coates 1968 | KZN | Oribi Gorge, rotting pineapple trash, 2.ii.1966 (T. J. Coates leg) AcV66/114 & 115, 10.xi.1965 and 11.x.1965, virgin soil, Empangeni (G. Nel leg) AcV 65/278 & 287 | ♂ holotype, two ♀ paratypes, two ♂ paratypes, all in the NIC, Department of Agricultural Technical Services, Pretoria |
| *Ectonura* sp. | Weiner & Najt 1991 | WC | Saasveld, near George, subtropical indigenous forest, on bark of *Olinia ventosa*, 23.xii.1986, V. Nicolai leg, 1 specimen | 1 specimen ISEA |
| *Friesea claviseta* Axelson, 1900 | Womersley 1934 | KZN | Pietermaritzburg, from dry leaves in garden, Sept 1951, R.F. Lawrence, 2 specimens |  |
| *Friesea claviseta* Axelson, 1900 | Womersley 1934 | WC | Stellenbosch, 12.xiii.1930, loose damp bark of fallen log (Womersley leg) |  |
| *Friesea versabilis* Barra, 1995 | Barra 1995 | KZN | Sodwana Bay, 5 cm depth, under pioneer vegetation, 03.ii.1992, J.-P. Rieb leg. | Holotype ♀, paratype ♂, RMCA |
| *Micranurida* sp. | Liu et al. 2012 | WC | Pine and Cape Flats Sand Fynbos litter, Tokai, July/August 2010 |  |
| *Najtafrica riebi* (Barra, 1994)* | Barra 1994 | KZN | Lake St Lucia, 100km south of Sodwana Bay, foot of first dune, moist sand at 5cm depth under pioneer vegetation, 6.ii.1992, J.-P. Rieb leg. | Holotype ♀ in RMCA, 3 ♀ paratypes (2 juvs) with author |
| *Neanura muscorum* (Templeton, 1835) | Coates 1968a | EC | Cathcart C.P. 15.v.1966, pine needle litter, T.J. Coates AcV 66/142. | NIC |
| *Neanura muscorum* (Templeton, 1835) (cf.) | Liu et al. 2012 | WC | Pine and Cape Flats Sand Fynbos litter, Tokai, July/August 2010 |  |
| *Paleonura* sp. | Liu et al. 2012 | WC | Pine litter, Tokai, July/August 2010 |  |
| *Pseudachorutella africana* Weiner and Najt, 1991 | Weiner & Najt 1991 | WC | Saasveld, near George, subtropical indigenous forest, pitfall traps, 17.xii.1986-17.i.1987, V. Nicolai leg. | Holotype ♂ (ISEA) |
| *Pseudachorutes alluaudi* (Delamare Deboutteville, 1946) | Paclt 1959 | KZN | National Park, Gudu Falls, under log in dense indigenous forest, alt about 6000ft, 4.iv.1951, SSAE leg, loc 206, Cathkin Peak, Jan. 1938 R.F. Lawrence leg, 1 specimen, Champagne Castle Hotel, January 1953 R. F. Lawrence leg., 7 specimens, - Ibidem January 1957, R.F. Lawrence leg, 4 specimens. | 5 specimens Museum Lund (Gudu Falls) |
| *Pseudachorutes univesicatus* Weiner and Najt, 1991 | Weiner & Najt 1991 | WC | Saasveld, near George, subtropical indigenous forest, pitfall traps, 17.xii.1986-17.i.1987, V. Nicolai leg. | Holotype ♀, paratypes 6 ♂, 2 ♀, 4 juv (ISEA), paratypes 4 ♂s, 2 ♀, 5 juv (MNHN) |
| *Vitronura joanna* (Coates, 1968) | Coates 1968a | NWP | Potchefstroom, 2.ii.1966, compost, J. Mathew, AcV 66/126. Pretoria, pot plant soil, 15.ix.1966, T. J. coats, AcV 66/157 | Holotype ♀, paratype ♂ and 8 ♀, 1 juv paratype, one ♀ paratype (NIC) |
| *Vitronura* sp. | Weiner & Najt 1991 | WC | Saasveld, near George, subtropical indigenous forest, on bark of *Scolopia mundii* 27.i.1987, V. Nicolai leg. | 1 specimen only (ISEA) |
| **Odontellidae** |  |  |  |  |
| *Odontella sylvatica* Weiner and Najt, 1991 | Weiner & Najt 1991 | WC | Saasveld, near George, subtropical indigenous forest, pitfall traps, 17.xii.1986-17.i.1987, V. Nicolai leg. | Holotype ♀, paratypes 7 (ISEA), 4 paratypes, MNHN |
| *Odontellina deharvengi* Barra, 1995 | Barra 1995 | KZN | St. Lucia, 100km south of Sodwana Bay, 5 cm below pioneer vegetation, 06.ii.1992, J.-P. Rieb leg. | Holotype ♀ and ♂ paratype (RMCA), two paratypes with author |
| *Superodontella empodialis* (Stach, 1934) | Paclt 1959 | KZN | Champagne Castle, Drakensberg, Feb 1946, R.F. Lawrence leg, 13 specimens |  |
| **Onychiuridae** |  |  |  |  |
| *Deuteraphorura inermis* (Tullberg, 1869) | Paclt 1959 | WC | Oranjezicht, Cape Town, 4 specimens | SA Museum |
| *Deuteraphorura inermis* (Tullberg, 1869) | Womersley 1934 | WC | Cape Town, under stones, 3 Aug and 6 Sep 1930 (Womersley leg) |  |
| *Orthonychiurus camerunensis* (Schött, 1926) | Paclt 1967 | G | Krugersdorp, Blyvooruitsig, damp garden soil, 29.ii.1956, 44 specimens AcV88, D.B. Wells leg | NIC |
| *Orthonychiurus saasveldensis* (Weiner and Najt, 1991) | Weiner & Najt 1991 | WC | Saasveld, near George, subtropical indigenous forest, on bark of *Scolopia mundii* 27.i.1987, V. Nicolai leg. | Holotype ♂, paratypes 1 ♀, 1 ♂, (ISEA), paratype 1 ♂ (MNHN) |
| *Orthonychiurus* sp. | Liu et al. 2012 | WC | Pine litter, Tokai, July/August 2010 |  |
| *Protaphorura armata* (Tullberg, 1869) | Lawrence 1953 | ? | No information given. |  |
| *Protaphorura matsumotoi* (Kinoshita, 1923) | Paclt 1959 | FS | Bloemfontein, under few pine trees, from soil containing large amounts of organic material, April 1957 (2 specimens), R. van Pletzen leg., Bloemfontein district, garden, from soil containing large amount of organic plant material, August 1957, R. van Pletzen leg, 41 specimens. |  |
| **Tullbergiidae** |  |  |  |  |
| *Delamarephorura capensis* Janion, Weiner & Deharveng, 2013 | Janion et al. 2013 | WC | Kleinmond, Betty’s Bay, sandy soil, Berlese extraction, coll. Louis Deharveng & Anne Bedos, (SAF-064), 11.ii.2008. | Holotype: 1 ♀ SAMC, Paratypes: 4 paratypes (1 ♂ and 3 juvs) in SAMC; 4  paratypes (1 ♂, 1 ♀ and 2 juvs) in MNHN; 3 paratypes  (2 ♀and 1 juveniles) in ISEA. |
| *Delamarephorura szeptyckii* Barra & Weiner, 2009 | Barra & Weiner 2009 | EC | Amatola mountains, Hogsback, dry prairie, 1600m a.s.l., August 1995, David Marshall leg. | Holotype ♀ and paratype juvenile MNHN, ♀ paratype ISEA |
| *Fissuraphorura miscellanea* Barra, 1995 | Barra 1995 | KZN | Sodwana Bay, 5 and 20 cm deep pioneer vegetation, 03.ii.1992, J.-P. Rieb leg. | All specimens ♀, holotype and paratype, RMCA |
| *Mesaphorura krausbaueri* (Börner, 1901) | Paclt 1959 | EC | Dordrecht district, small poplar grove from soil containing large amount of decomposed material (about 280 specimens), July and October 1957, R. van Pletzen leg. |  |
| *Mesaphorura krausbaueri* (Börner, 1901) | Paclt 1959 | FS | Bloemfontein, garden, from soil containing large amount of organic plant material, August 1957, R. van Pletzen (about 250 specimens) |  |
| *Mesaphorura krausbaueri* (Börner, 1901) | Womersley 1934 | WC | Hout Bay under stones 4.viii.1930, Cape Town 19.viii.1930, Stellenbosch 18.viii.1930 (Womersley leg) |  |
| *Mesaphorura macrochaeta* Rusek, 1976 (cf.) | Liu et al. 2012 | WC | Pine and Cape Flats Sand Fynbos litter, Tokai, July/August 2010 |  |
| *Mesaphorura yosii* (Rusek, 1967) | Barra 1995 | KZN | Sodwana Bay |  |
| *Paratullbergia callipygos* (Börner, 1902) | Womersley 1934 | WC | Cape Town, August 1930 (Womersley leg) |  |
| *Tullbergia kilimanjarica* (Delamare Deboutteville, 1953) | Paclt 1959 | KZN | Champagne Castle, Drakensberg, Feb 1946, R.F. Lawrence leg, ,8 specimens, Ibidem, under stones embedded in damp humus in the forest floor, with Eukoenenia and Pauropods, at about 5500 ft., April 1957, R. F. Lawrence leg, 3 specimens, Pietermaritzburg, from dry leaves in garden, Spet 1951, R. F. Lawrence leg. (6 specimens), Bisley, Pietermaritzburg, Oct 1955, R. F. Lawrence leg. (10 specimens) |  |
| *Tullbergia kilimanjarica* (Delamare Deboutteville, 1953) | Coates 1970 | MP | Kruger National Park, under dead leaves, Klopperfontein No. 15 |  |
| *Tullbergia kilimanjarica* (Delamare Deboutteville, 1953) | Paclt 1959 | WC | Cape Peninsula, Cape Point Nature Reserve, sieved from wet leaves in low bush, 10.xii.1950 Loc. 79, Hout Bay, Skoorsteenkop, sieved from vegetable debris in dense indigenous forest, 600 ft., 28.i.1951, loc. 161all SSAE leg. | 1 specimen, 2 Museum Lund |
| *Tullbergia kilimanjarica* (Delamare Deboutteville, 1953) | Paclt 1967 | WC | van Rhynsdorp, Knersvlakte, 3 miles from van Rhyns pass, 22.iii.1956, 1 specimen AcV93, D.v.V. Webb leg. | NIC |
| *Tullbergia meridionalis* Cassagnau & Rapoport, 1962 | Barra 1995 | KZN | St. Lucia, 100km south of Sodwana Bay, wet sand at foot of dune, 06.ii.1992, J.-P. Rieb leg. |  |
| *Tullbergia* sp. | Liu et al. 2012 | WC | Pine and Cape Flats Sand Fynbos litter, Tokai, July/August 2010 |  |
| **ENTOMOBRYOMORPHA** |  |  |  |  |
| **Isotomidae** |  |  |  |  |
| *Archisotoma sabulosa* Barra, 1997 | Barra 1997 | KZN | Sand under pioneer plants 5 cm deep, 03.ii.1992, J.-P. Rieb leg. | Holotype ♀, one paratype and two other specimens, MNHN |
| *Arlea tridens* Barra, 1997 | Barra 1997 | KZN | Sodwana Bay National park, litter and superficial humus of dunes next to dense forest, 02.ii.1992, J.-P. Rieb leg. | Holotype ♀, one paratype and two other specimens, MNHN |
| *Ballistura schoetti* (Dalla Torre, 1895) | Paclt 1967 | EC | Graaff-Reinet 29.ix.1939, D.v.V. Webb leg. | 23 specimens NIC |
| *Ballistura schoetti* (Dalla Torre, 1895) | Paclt 1959 | WC | Cape Flats, 1 M east of Zeekoeivlei, among dense vegetation at freshwater pool, 8.12.1950, SSAE leg., loc 76. | 1 specimen Museum Lund |
| *Ballistura schoetti* (Dalla Torre, 1895) | Paclt 1967 | WC | Mamre Road, Waterkloof Farm, July 1937, D.v.V. Webb leg. | 14 specimens NIC |
| *Ballistura schoetti* (Dalla Torre, 1895) | Womersley 1934 | WC | Stellenbosch, 28.viii.1927 Dr Hesse leg. |  |
| *Ballistura schoetti* (Dalla Torre, 1895) | Yosii 1959 | WC | Beach of Rondevlei Marsh, 10.iii.1958, 25 specimens. |  |
| *Clavisotoma africana* (Womersley, 1934) | Paclt 1959 | WC | Cape Peninsula, Cape Point Nature Reserve, sieved from wet leaves in low bush, 10.xii.1950 SSAE leg. Loc. 79. | 8 specimens Museum Lund |
| *Clavisotoma africana* (Womersley, 1934) | Womersley 1934 | WC | Rain pools in Kimberley, Feb 1915 Miss Witman leg, rain pools Cape Town June 1915 K.H. Barnard leg. | Co-types in the SAMC |
| *Cryptopygus* sp. 1 | Liu et al. 2012 | WC | Pine and Cape Flats Sand Fynbos litter, Tokai, July/August 2010 |  |
| *Cryptopygus* sp. 2 | Liu et al. 2012 | WC | Pine and Cape Flats Sand Fynbos litter, Tokai, July/August 2010 |  |
| *Folsomides americanus* Denis, 1931 | Barra 1997 | KZN | Sodwana Bay |  |
| *Folsomides americanus* Denis, 1931 | Paclt 1959 | KZN | Champagne Castle, Drakensberg, Feb 1946 (3 specimens), Pietermaritzburg, garden, from dry leaves, Sept 1951 (1 specimen), all R.F. Lawrence leg. |  |
| *Folsomina onychiurina* Denis, 1931 | Barra 1997 | KZN | Sodwana Bay |  |
| *Hemisotoma thermophila* (Axelson, 1900) | Womersley 1934 | KZN | Inchanga, Nov. 1917, K.H. Barnard leg. | 1 young, poorly preserved specimen (noted by Paclt 1959) |
| *Hemisotoma thermophila* (Axelson, 1900) | Coates 1970 | WC | Tsitsikama Forest and Coastal National Park, under fallen oak leaves, near conservator's house and under rotting leaves, Storm River Mouth. |  |
| *Hemisotoma thermophila* (Axelson, 1900) | Paclt 1959 | WC | Border 20 miles N of Matatiele (S. of Qacas Nek), 6500 ft. alt., at small rock pool, 08.iii.1951, loc 214 SSAE leg. | 1 specimen Museum Lund |
| *Isotoma finitima* Scherbakov, 1899 | Paclt 1959 | KZN | Champagne Castle, Drakensberg, Feb 1946, R. F. Lawrence leg (29 specimens). |  |
| *Isotoma mauretanica* Handschin, 1926 | Paclt 1959 | WC | Table Mountain, Stellenbosch (from Womersley 1934). | 2 specimens in SAMC |
| *Isotoma mauretanica* Handschin, 1926 | Womersley 1934 | WC | Table Mountain Cape Town 4 June 1913 (K.H. Barnard leg), Stellenbosch 7.vii.1930 (Womersley leg). | Two specimens observed by Paclt 1959 in SAMC, observed to be *Isotomurus palustris,* but lack trichobothria*.* |
| *Isotomiella sodwana* Barra, 1997 | Barra 1997 | KZN | Sodwana Bay National Park, litter and humus on surface of sand dunes, 02.ii.1992, J.-P. Rieb leg. | Two copies of holotype (sex not visible), MNHN |
| *Isotomodes productus* (Axelson, 1906) | Womersley 1934 | WC | Signal Hill, Cape Town, under stones, 31.viii.1930 (Womersley leg). |  |
| *Isotomurus balteatus* (Reuter, 1876) | Womersley 1934 | WC | Table Mountain Cape Town, 12.ix.1913 K.H. Barnard leg, Stellenbosch 29.viii.1927 Dr, Hesse leg. |  |
| *Isotomurus maculatus* (Schäffer, 1976) (cf.) | Liu et al. 2012 | WC | Pine litter, Tokai, July/August 2010 |  |
| *Isotomurus palustris* (Müller, 1776) | Paclt 1967 | EC | Uitenhage, Prickly Pear Laboratory, on banks of Swartkops River, 3.xi.1938, 1 specimen Ac61, Abderdeen, 21.xi,1939, 15 specimens AcV68, all D.v.V. Webb leg. | NIC |
| *Isotomurus palustris* (Müller, 1776) | Paclt 1967 | G | Pretoria, Parktown, grass on banks of Apies River, 24.ix.1937, 1 specimen, AcV45 D.v.V. Webb leg. |  |
| *Isotomurus palustris* (Müller, 1776) | Paclt 1967 | KZN | Mkuzi Game Reserve, Zululand, light traps, D. v. V. Webb leg, December 1945 to January 1946, 1 specimen AcV82. | NIC |
| *Isotomurus palustris* (Müller, 1776) | Paclt 1967 | WC | Mamre Road, Waterkloof Farm, 3.vii 1937, 6 specimens AcV28; Gordon's Bay, Steenbras River Mouth, moss and rotten leaves, 1.viii.1937, 1 specimen AvV31, 4.viii.1937, 7 specimens AcV34 and 11.viii.1937, 17 specimens AcV40; Somerset West, Sit Lowrys Pass, 12.viii.1937, 1 specimen, AcV41; gordons Bay, 5.viii.1938, 20 specimens AcV55, all D.v.V. Webb leg. | NIC |
| *Isotomurus palustris* (Müller, 1776) | Womersley 1934 | WC | Ceres, Oct 1927 (K.H. Barnard leg). |  |
| *Isotomurus palustris* (Müller, 1776) (cf.) | Liu et al. 2012 | WC | Pine litter, Tokai, July/August 2010 |  |
| *Isotomurus tricuspis* Börner, 1906 | Paclt 1959 | WC | Stellenbosch and Table Mountain; 3 juvenile specimens from Rondebosch, 29.vii.1930, H. Womersley leg. | from slide in SAM, 1 specimen |
| *Isotomurus tricuspis* Börner, 1906 | Paclt 1967 | WC | Table Mountain Drive Waterfall no. 2, damp moss, 5.v.1956, 2 specimens AcV106, D.v.V. Webb leg. | NIC |
| *Micranurophorus musci* Bernard, 1977 | Barra 1997 | KZN | Sodwana Bay, humid sand 20 cm deep under pioneer vegetation, 06.ii.1992, J.-R. Rieb leg. |  |
| *Mucrosomia caeca* (Wahlgren, 1906) | Paclt 1959 | KZN | Champagne Castle, Drakensberg, Feb 1946 (2 specimens), R. F. Lawrence leg. |  |
| *Mucrosomia caeca* (Wahlgren, 1906) | Paclt 1959 | WC | Cape Peninsula, Table Mountain, Wynberg Cave Ravine, sieved from wet vegetable debris, alt 2400 ft., 18.xii.1950, SSAE leg., loc. 84 | 2 specimens Museum Lund |
| *Mucrosomia caeca* (Wahlgren, 1906) (cf.) | Liu et al. 2012 | WC | Pine and Cape Flats Sand Fynbos litter, Tokai, July/August 2010 |  |
| *Parisotoma mossopi* (Womersley, 1934) | Paclt 1959 | FS | Bloemfontein, under few pine trees, from soil containing large amounts of organic material, April 1957 (1 specimen), R. van Pletzen leg. |  |
| *Parisotoma notabilis* (Schäffer, 1896) | Paclt 1959 | WC | Cape Peninsula, Cape Point Nature Reserve, sieved from wet leaves in low bush, 10.xii.1950 SSAE leg. Loc. 79 (2 specimens), Table Mountain, Wynberg Cave Ravine, sieved from wet vegetable matter, alt 2400 ft., 18.xii.1950, SSAE leg. Loc 84 (69 specimens)., Houtbay, Skoorsteenkop, sieved from vegetable debris in dense indigenous forest, alt 600 ft., 18.i.1951, loc 161 (1 specimen). | Museum Lund |
| *Parisotoma notabilis* (Schäffer, 1896) | Paclt 1967 | WC | Van Rhynsdorp Knersvlakte, 3 miles form Van Rhyns Pass, 22.iii.1956, 17 specimens AcV93; Hout Bay, Chapmans Peak Drive, damp soil and moss, 22.iv.1956, 1 specimen AcV102, Table Mountain Drive, humus and pine needles, 5.v.1956, 13 specimen AcV105, Idem, Table Mountain Drive waterfall no. 2, damp moss, 5.v.1956, 10 specimens AcV106, all material D.v.V. Webb leg. | NIC |
| *Parisotoma obscurocellata* Potapov, Janion and Deharveng, 2011 | Potapov, Janion & Deharveng 2011 | WC | Type locality: Western Cape, South Africa, Betty's Bay, 11.iii.2008, under creeping plants, extraction in Berlese funnels, legs. L. Deharveng and A. Bedos, SAF063. | Holotype ♀ on slide and about 60 paratypes (12 on slide, 48 in alcohol), holotype and 20 paratypes kept at SAMC and 20 paratypes in MNHN |
| *Parisotoma sexsetosa* Potapov, Janion and Deharveng, 2011 | Potapov et.al. 2011 | WC | Type locality: Table Mountain National Park, 10.iii.2009, native forest, sieving and extraction from Berlese funnel, near Wynberg cave, SAF141m L. Deharveng & A. Bedos leg, RSA09_TBM001 C. Janion leg., near inchuk cave entrance SAF144 (LD & AB). | Holotype ♀, about 1560 paratypes, Holotype ♀ and 140 paratypes SAMC, 140 paratypes in MNHN, 140 paratypes in MSPU |
| *Parisotoma* sp. 1 | Liu et al. 2012 | WC | Pine and Cape Flats Sand Fynbos litter, Tokai, July/August 2010 |  |
| *Parisotoma* sp. 2 | Liu et al. 2012 | WC | Pine and Cape Flats Sand Fynbos litter, Tokai, July/August 2010 |  |
| *Pauropygus caussaneli* (Thibaud, 1996) | Barra 1997 | KZN | Sodwana Bay, sand under pioneer plants 5 cm deep, 03.ii.1992, J.-P. Rieb leg. | Holotype ♀ paratype and two other preparations, MNHN |
| *Proisotoma davidi* Barra, 2001 | Barra 2001 | EC | Amatola Mountains, Themeda grassland soils at Hogsback, 1600 m a.s.l., David Marshall leg. | Holotype ♀ and three paratypes, MNHN |
| *Proisotoma minuta* (Tullberg, 1871) | Paclt 1959 | EC | Dordrecht district, from heap of decomposing mealie cobs, July and October 1957, R. van Pletzen leg, about 60 specimens. |  |
| *Proisotoma minuta* (Tullberg, 1871) | Paclt 1967 | EC | Queenstown, 22.iii.1939, A number of body wracks AcV67. | NIC |
| *Proisotoma minuta* (Tullberg, 1871) | Paclt 1959 | FS | Bloemfontein, under few pine trees, from soil containing large amounts of organic material, April 1957 (1 specimen), R. van Pletzen leg., Bloemfontein district, garden, from soil containing large amount of organic plant material, August 1957, R. van Pletzen leg. (4 specimens). |  |
| *Proisotoma minuta* (Tullberg, 1871) | Paclt 1959 | KZN | Pietermaritzburg, from dry leaves in garden, Sept 1951, R.F. Lawrence, 6 specimens. |  |
| *Proisotoma minuta* (Tullberg, 1871) | Paclt 1959 | WC | Cape Peninsula, Cape Point Nature Reserve, sieved from wet leaves in low bush, 10.xii.1950 Loc. 79, SSAE leg. | 30 specimens Museum Lund |
| *Proisotoma minuta* (Tullberg, 1871) | Paclt 1967 | WC | Simonstown, Red Hill Drive, damp soil and moss, 15.iv.1956, 1 specimen AcV101, D,v.V. Webb leg, Idem, Municipal Centre, soil in colony of *Coptotermes formosanus* Shiraki in the Termite Research Laboratory of the Division of Entomology, Simonstown, 9.viii.1956, 1 specimen, AcV107, Mrs P.E. de Wet leg. | NIC |
| *Subisotoma* sp. | Liu et al. 2012 | WC | Pine litter, Tokai, July/August 2010 |  |
| **Entomobryidae** |  |  |  |  |
| *Capbrya marshalli* Barra, 1999* | Barra 1999 | EC | Hogsback, Amatola Mountains, 1600m a.s.l. in Themeda grassland, 40 km from University of Alice | Holotype ♀ and three paratypes ISNB |
| *Capbrya themeda* Barra, 1999* | Barra 1999 | EC | Hogsback, Amatola Mountains, 1600m a.s.l. in Themeda grassland, 40 km from University of Alice | Holotype ♀ and two paratypes ISNB |
| *Coecobrya caeca* (Schött, 1896) | Goto 1953 | WC | Cango Caves, nr Oudtshoorn (J.S. Harington & I.J. Lewis leg) - see Harington (1952) - in abundance in first mile and half of cave in rotting wood, moss, on rocks and stalagmites, and in very high numbers on floor of main chamber on bat guano. | Deposited in SAMC, also possession of collectors and author |
| *Coecobrya hoefti* (Schäffer, 1896) | Paclt 1959 | WC | Groot Swartberg Mountain Range, Cango Caves, 19.x.1948 (3 specimens), B. Hanstrom leg, - ibidem in central part of cave, 6.1.1951 (1 specimen), SSAE leg. Loc 121. | Museum Lund |
| *Entomobrya atrocincta* Schött, 1897 | Paclt 1967 | WC | Paarl, fallen leaves, 29.viii.1956, 7 specimens AcV29, D.v.V. Webb leg | NIC |
| *Entomobrya decemfasciata* (Packard, 1873) | Womersley 1934 | WC | Muizenberg 25 July 1930 Womersley leg. |  |
| *Entomobrya lanuginosa (*Nicolet, 1842) | Womersley 1934 | WC | Rondebosch, Cape Town, 29.vii.1930 Womersley leg. |  |
| *Entomobrya minima* Brown, 1926 | Brown 1926 | KZN | Umkomaas, under a stone, 16.vii.1917, P.A. Boxton leg. | not mentioned |
| *Entomobrya multifasciata* (Tullberg, 1871) | Paclt 1967 | G | Pretoria, Parktown, grass on banks of Apies River, 24.ix.1937, 2 specimens, AcV45 D.v.V. Webb leg | NIC |
| *Entomobrya multifasciata* (Tullberg, 1871) | Paclt 1967 | NC | Upington, banks of Orange River, Dec 1937, 3 specimens, AcV48, D.v.V. Webb leg | NIC |
| *Entomobrya nicoleti (*Lubbock, 1876) | Womersley 1934 | WC | Stellenbosch, 28.viii.1927 Dr. Hesse leg., Rondebosch, Cape Town, 29.vii.1930 Womersley leg. |  |
| *Entomobrya nivalis* (Linnaeus, 1758) | Paclt 1959 | EC | Dordrecht district, from heap of decomposing mealie cobs (21 specimens), July and October 1957, R. van Pletzen leg. (1 specimen) |  |
| *Entomobrya nivalis* (Linnaeus, 1758) | Paclt 1959 | FS | Fouriesburg District, from surface of rainwater pool, January or April 1957, R. van Pletzen leg. (1 specimen) |  |
| *Entomobrya nivalis* (Linnaeus, 1758) | Paclt 1967 | KZN | New Hanover, Ashenden Estates, on exterior of the wattle bagworm *Kotochalia junodi,* 21.viii.1947, 1 specimen AcV85, D.v.V. Webb leg. | NIC |
| *Entomobrya nivalis* (Linnaeus, 1758) | Coates 1970 | WC | Tsitsikama Forest and Coastal NP, from rotting leaves in indigenous forest, Storms River |  |
| *Entomobrya nivalis* (Linnaeus, 1758) | Paclt 1959 | WC | Tsitsikama Forest, Storms river, 12.i.1951, SSAE leg, loc. 134 (5 specimens), Cape Peninsula, Cape Point Nature Reserve, sieved from wet sieved on low bush, 10.xii.1950, SSAE leg., loc. 79 (3 specimens), Groot Swartberg Mountain Range, Cango caves, 19.x.1948 (2 specimens), B. Hanstrom leg. | Museum Lund |
| *Entomobrya* *nivalis* Linnaeus, 1758 (cf.) | Yosii 1959 | WC | Kloof nek, 5.xii.1957, 4 specimens | ♀ of young stage, exact identification is not possible |
| *Entomobrya* sp. | Lawrence 1953 | ? | No information given |  |
| *Entomobrya* sp. | Paclt 1967 | WC | Gordons Bay, grass near sea, 4.viii.1937, 6 specimens, AcV32, Somerset West, 4.viii.1937, 1 specimen AcV33; Gordons bay, 10.viii.1937, 1 specimen, AcV38, all D.v.V. Webb leg. | NIC |
| *Entomobrya* sp.1 | Liu et al. 2012 | WC | Pine and Cape Flats Sand Fynbos litter, Tokai, July/August 2010 |  |
| *Entomobrya* sp.2 | Liu et al. 2012 | WC | Cape Flats Sand Fynbos litter, Tokai, July/August 2010, one specimen |  |
| *Entomobrya* sp.3 | Liu et al. 2012 | WC | Cape Flats Sand Fynbos litter, Tokai, July/August 2010 |  |
| *Entomobrya* sp.4 | Liu et al. 2012 | WC | Pine litter, Tokai, July/August 2010 |  |
| *Lepidocyrtus cyaneus* Tullberg, 1871 | Paclt 1959 | EC | Dordrecht district, small poplar grove from soil containing large amount of decomposed material (2 specimens), October 1957, R. van Pletzen leg. |  |
| *Lepidocyrtus cyaneus* Tullberg, 1871 | Paclt 1959 | KZN | Pietermaritzburg, dry leaves in garden, R.F. Lawrence leg., Sept 1951 (1 specimen), Drakensberg, about 13 miles ENE from Rhodes, among stones on mountain slope, at small stream, 8000 ft., 9.iii.1951, SSAE leg, Loc. 219 | 1 specimens Museum Lund (SSAE leg) |
| *Lepidocyrtus ferrugineus* (Schött, 1893) | Paclt 1959 | KZN | Pietermaritzburg, dry leaves in garden, R.F. Lawrence leg., Sept 1951 (120 specimens) |  |
| *Lepidocyrtus lanuginosus* (Gmelin, 1788) | Paclt 1967 | WC | Table Mountain Drive, humus and pine needles, 5.v.1956, 7 specimens, AcV105, D.v.V. Webb leg | NIC |
| *Lepidocyrtus lanuginosus* (Gmelin, 1788) | Womersley 1934 | WC | Franschhoek 200 feet, Dec. 1926 (K.H. Barnard leg), Stellenbosch 12.viii.1930, Rondebosch 19.vii.1930, Cape Town 24.viii.1930, Signal Hill 31.viii.1930, Hout Bay 30.viii.1930, Kirstenbosch 2.viii.1930 (all Womersley leg.) |  |
| *Lepidocyrtus* *lanuginosus* (Gmelin, 1788) (cf.) | Paclt 1967 | WC | Mamre Road, Waterkloof Farm, 3.vi.1937, 3 specimens AcV28; Gordons Bay, 4.viii.1937, 2 specimens AcV34, 11.viii.1937, 2 specimens, AcV40 and 5.vii.1938, 7 specimens AcV55; Velddrift, damp soil along banks of berg River, 20.iii.1956, 1 specimenAcV90; Hout Bay Chapmans Peak Drive, damp soil and moss, 22.iv.1956, 1 specimen AcV102, all D.v.V. Webb leg. | NIC |
| *Lepidocyrtus lanuginosus* (Gmelin, 1788) (cf.) | Yosii 1959 | WC | Table Mountain, 10 specimens, 5.xii.1957 |  |
| *Lepidocyrtus* sp | Lawrence 1953 | ? | No information given. |  |
| *Lepidocyrtus* sp. 1 | Liu et al. 2012 | WC | Pine and Cape Flats Sand Fynbos litter, Tokai, July/August 2010 |  |
| *Lepidocyrtus* sp. 2 | Liu et al. 2012 | WC | Pine litter, Tokai, July/August 2010 |  |
| *Lepidokrugeria meyerae* Coates, 1969* | Coates 1969 | MP | Letaba River, Kruger National Park, N237, TvL., dead leaves, 14.i.1964, T. J. Coates AcV64/94 and under *Protulacaria afra*, Shingwidzi Rest Camp (Coates 1970) | Holotype, 3 paratypes from KNP, NIC, not listed, 30 other specimens from KNP, Groblersdal and Machava, Mozambique. |
| *Orchesella hexfasciata* Harvey, 1896 | Paclt 1959 | FS | Bloemfontein District, in garden, from soil containing large amounts of plant material, Aug 1957, R. van Pletzen leg, (1 specimen) |  |
| *Orchesella hexfasciata* Harvey, 1896 | Paclt 1959 | G | Johannesburg, Ventersdorp Gold Mine, in decaying timber, June 1943, Miss. D. Weintroub leg. (32 specimens). |  |
| *Pseudosinella alba* (Packard, 1873) | Paclt 1959 | EC | Dordrecht district, from heap of decomposing mealie cobs (7 specimens), July and October 1957, R. van Pletzen leg., also small poplar grove from soil containing decomposing leaves (21 specimens) |  |
| *Pseudosinella alba* (Packard, 1873) | Paclt 1959 | WC | Cape Peninsula, Cape Point Nature Reserve, sieved from wet leaves in low bush, 10.xii.1950 SSAE leg. Loc. 79 | 8 specimens Museum Lund |
| *Pseudosinella biguttata* Barra 1997 | Barra 1997 | KZN | Sodwana Bay National Park, superficial litter and humus on dunes, sandy forest bush shrub, 02.ii.1992, J.-P. Rieb leg. | Holotype ♀, one paratype and one other example, MNHN |
| *Pseudosinella* *candida* Folsom, 1902 (cf.) | Yosii 1959 | WC | Kloof Nek, 5.xii.1957, 31 specimens |  |
| *Pseudosinella immaculata* (Lie-Pettersen, 1897) | Paclt 1959 | KZN | Champagne Castle, Drakensberg, Feb 1946, R.F. Lawrence leg. (7 specimens) |  |
| *Pseudosinella octopunctata* Börner, 1901 | Paclt 1959 | FS | Bloemfontein, under few pine trees, from soil containing large amounts of organic material, April 1957 (1 specimen) |  |
| *Pseudosinella octopunctata* Börner, 1901 | Paclt 1959 | WC | Table Mountain, Wynberg Cave Ravine, sieved from wet vegetable debris, alt 2400ft, 18.xii.1950, SSAE leg. Loc. 84 | 6 specimens Museum Lund |
| *Pseudosinella* sp. | Liu et al. 2012 | WC | Pine and Cape Flats Sand Fynbos litter, Tokai, July/August 2010 |  |
| *Seira addoensis* Coates, 1968 | Coates 1968 | EC | Holotype from under grass, Addo Elephant NP, 12.i.1965 AcV65/98; 2 paratypes one from soil andother one from *Euclea undulata* Addo Elephant NP, 11.i.1965 AcV65/46 & 47. Several other specimens, but with incomplete antennae, all T.J. Coates leg. | Holotype and 2 paratypes, NIC |
| *Seira anncla* Coates, 1968 | Coates 1968 | EC | Grahamstown district; holotype and 1 paratype from *Scutia myrtina* (Burm. F) Kurz and 4 paratypes from Gewsia sp, 13.i.1965, T.J. Coates leg, AcV65/67, 69, 70. About 40 other specimens from the Eastern Cape. | Holotype and 5 paratypes, NIC |
| *Seira anncla* Coates, 1968 | Coates 1970 | WC | Tsitsikama Forest and Coastal NP, on grass next to sea, Mountain Zebra NP, on unidentified shrub. |  |
| *Seira annulicornis* (Börner, 1903) | Coates 1968 | FS | Wide distribution |  |
| *Seira annulicornis* (Börner, 1903) | Coates 1968 | G | Wide distribution |  |
| *Seira annulicornis* (Börner, 1903) | Coates 1968 | KZN | From north-east of a line joining Pongola and Pretoria, incl. Mozambique, about 250 specimens. |  |
| *Seira annulicornis* (Börner, 1903) | Coates 1970 | MP | Kruger National Park, Klopperfontein, in soil around pool. |  |
| *Seira annulicornis* (Börner, 1903) | Yosii 1959 | WC | Table Mountain, 8 specimens, 11.iii.1958 |  |
| *Seira annulipes* (Handschin, 1929) | Womersley 1934 | KZN | Inchanga, Nov 1917 (K.H. Barnard leg) | 24 specimens in SAMC (according to Coates 1968) |
| *Seira annulipes* (Handschin, 1929) | Womersley 1934 | WC | Keeromberg, Worcester Mountains, 3500 feet, Sept 1930 (K.H. Barnard leg) |  |
| *Seira annulosa (*Wahlgren, 1906) | Womersley 1934 | WC | Muizenberg, shore herbage, 25.vii.1930 (Womersley leg) |  |
| *Seira barnardi* (Womersley, 1934) | Paclt 1967 | NWP | Marico District, fallen leaves, 13.i.1930, one specimen AcV3, J.C. Faure leg | NIC |
| *Seira barnardi* (Womersley, 1934) | Coates 1968 | WC | Suurbraak, Swellendam and Tsitsikama District (T.J. Coates leg) |  |
| *Seira barnardi* (Womersley, 1934) | Coates 1970 | WC | Tsitsikamma Forest and Coastal NP, on *Passerinaxsp.* |  |
| *Seira barnardi* (Womersley, 1934) | Paclt 1959 | WC | Cape Peninsula, Cape Point Nature Reserve, sieved from wet leaves in low bush, 10.xii.1950, loc. 79 (12 specimens), Table Mountain, Wynberg Cave Ravine, sieved from wet debris, 2400ft, 18.xii.1950 loc 84 (32 specimens), Hout Bay, Skoorsteenkop, sieved from vegetable debris in dense indigenous forest, 600ft, 28.i.1951, loc.161 (15 specimens), all SSAE leg. | Museum Lund |
| *Seira barnardi* (Womersley, 1934) | Paclt 1967 | WC | Port St. Johns, 14.i.1931, 4 specimens, AcV4, W. Powell leg | NIC |
| *Seira barnardi* (Womersley, 1934) | Womersley 1934 | WC | Cape Town 16.xii.1916, 1.viii.1915 1000 feet (K.H. Barnard leg), Kirstenbosch 22.vii.1930 (Womersley leg) | Co-types in SAMC (slide 73, 75 and 76). |
| *Seira barnardi (*Womersley, 1934) | Yosii 1959 | WC | Cape Town, Kirstenbosch, 47 specimens, 10.iii.1938 (1958?) |  |
| *Seira barnardi* (Womersley, 1934) (cf.) | Liu et al. 2012 | WC | Pine litter, Tokai, July/August 2010 |  |
| *Seira capensis* (Womersley, 1934) | Coates 1968 | EC | Port Alfred (T.J. Coates leg), 166-167. |  |
| *Seira capensis* (Womersley, 1934) | Coates 1968 | WC | Citrusdal and Tulbagh (T.J. Coates leg), 166-167. |  |
| *Seira capensis* (Womersley, 1934) | Womersley 1934 | WC | Type locality: Matroosberg 3500 feet Jan 1917 (Ceres side, Farm Laken Vallei, K.H. Barnard leg) | Co-types in SAMC (slide 82 and 83 according to Coates 1968) |
| *Seira capensis* (Womersley, 1934) | Yosii 1959 | WC | ♀ specimen from Porcupine Buttress 11.iii.1958, ♂ from Kloofnek 6.xii.1957 |  |
| *Seira damerella* Coates, 1968 | Coates 1968 | L | Holotype and 1 paratype from grass under citrus, Politsi, 16.i.1964, 1 paratype from under dead leaves at Letsitele, 1 from grass at Phalaborwa, both 16.i.1964... About 20 specimens from Lowveld, all T.J. Coates leg. | Holotype, NIC |
| *Seira damerella* Coates, 1968 | Coates 1968 | MP | One paratype from mulch in banana orchard, Nelspruit, 7.vii.1966, T.J. Coates leg. |  |
| *Seira damerella* Coates, 1968 | Coates 1970 | MP | Kruger National Park, under dead leaves, Letaba River, No. 237 and under *Hyphaene crinita*, Shingwidzi Rest Camp. |  |
| *Seira dayi* Yosii, 1959 | Coates 1968 | WC | 10 specimens from Grootvadersbosch and Heidelberg, T.J. Coates leg |  |
| *Seira dayi* Yosii, 1959 | Yosii 1959 | WC | Type locality: Skeleton Gorge, cape Town, 3 specimens, 6.xii.1957 |  |
| *Seira eleana* Coates, 1968 | Coates 1968 | MP | Holotype and 4 paratypes from dry grass, Bundu Inn Groblersdal district, 15.iii.1967, AcV 67/14. About 100 other specimens from the Kruger NP and Mozambique. | Holotype, NIC |
| *Seira eleana* Coates, 1968 | Coates 1968, 1970 | MP | Kruger National Park, under dead leaves, No. 41; under rotting leaves, Shingwidzi Rest Camp; from soil, Klopperfontein; under elephant droppings, Shingwidzi Rest Camp; under dead leaves, Letaba River, No 237 and also no. 216. |  |
| *Seira flavovirens* (Börner, 1903) | Coates 1968 | WC | Elsenberg, Trawal |  |
| *Seira flavovirens* (Börner, 1903) | Womersley 1934 | WC | Fransch hoek 200 feet Dec1916, Matroosberg Ceres side, Farm Laken Vallei 3500 feet Jan 1917 (K.H. Barnard leg), Hout Bay Aug 1930 (Womersley leg) | 2 specimens in SAMC according to Coates (1968) |
| *Seira flavovirens* (Börner, 1903) | Yosii 1959 | WC | Porcupine Buttress, 2 specimens, 8.iii.1958 |  |
| *Seira grisea* (Womersley, 1934) | Coates 1968, 1970 | WC | 2 specimens from *Hedera helix,* Katberg, 15.i.1965, AcV65/74, and 2 specimens from *Elytropappus rhinocerotis* Bontebok National Park, Swellendam, 7.ii.1967, AcV 66/167, all T.J. Coates leg. | NIC |
| *Seira grisea* (Womersley, 1934) | Womersley 1934 | WC | Rosebank, Cape Town 22.vii.1930, Kirstenbosch 23.vii.1930 Womersley leg. | Lectotype and paralectotype SAMC, re-description by Coates 1968 based on slide 71 (remounted) |
| *Seira grisea annulata* Womersley, 1934 | Womersley 1934 | WC | Kirstenbosch, 23.vii.1930 Womersley leg. | Co-types in SAMC |
| *Seira incerta* (Handschin, 1926) | Womersley 1934 | WC | Keurbooms Estuary, Jan. 1916 (K.H. Barnard leg) |  |
| *Seira laeta* (Börner, 1908) | Börner 1908 | NC | Port Nolloth, April 1905, 3 specimens, L. Schultze leg. |  |
| *Seira lindei* Coates, 1968 | Coates 1968 | EC | Type locality: Holotype and 2 paratypes from rotting leaves in stream, Mountain Zebra NP, 29.i.1965 AcV65/28; 2 paratypes from rotting grass, Zuurberg, 13.i.1965 AcV65/65; other specimens from Addo, Fort Beaufort, van Stadens River, and , all T.J. Coates leg. | Holotype, NIC |
| *Seira lindei* Coates, 1968 | Coates 1968 | WC | Heidelberg, Piekenierskloof, T.J. Coates leg. |  |
| *Seira marephila* Coates, 1968 | Coates 1968 | EC | 30 other specimens from coast between East London and Mossel Bay, all T.J. Coates leg. |  |
| *Seira marephila* Coates, 1968 | Coates 1968 | WC | Type locality: Holotype from *Zygophyllumxsp.*Hartenbos, 25.i.1965 AcV65/32; 2 paratypes from rotting leaves, Storms River Mouth, 20.i.1965, 3 paratypes from *Rhus undulata, Serruria fucifolia* and under rotting wood respectively, Bontebok NP, 7 and 8.ii.1967 AcV66/169, 171 and 172. 30 other specimens from coast between East London and Mossel Bay, all T.J. Coates leg. | Holotype, NIC |
| *Seira mathewsi* Coates 1968 | Coates 1968, 1970 | EC | 1 paratype from *Grewia* sp, Mountain Zebra National Park, 27.i.1965, AcV65/37, 2 paratypes from grass in vlei, Joubertina, 16.i.1965, AcV65/36 (all T.J.Coates leg). | 3 paratypes, NIC |
| *Seira mathewsi* Coates, 1968 | Coates 1968, 1970 | WC | Type locality: Holotype from *Serruria fucifolia* and 3 paratypes from *Chondropetalum nudum* Bontebok National Park, Swellendam, 8.ii.1967, AcV67/172 & 173, other specimens from Grootvadersbosch (all T.J.Coates leg). | Holotype, NIC |
| *Seira metala* Coates, 1968 | Coates 1968 | WC | Type locality: Grootvadersbosch, Swellendam District, holotype and 2 paratypes from under decaying bluegum leaves, 9.ii.1967 T.J. Coates leg, AcV66/182 | Holotype and 2 paratypes, NIC |
| *Seira metarsiosa* Coates, 1968 | Coates 1968 | FS | Type locality: Holotype and 3 paratypes from grass, Hammonia district, 16.viii.1967, T.J. Coates leg, AcV67/49 ... about 65 other specimens from Edenville, Ficksburg and Clocolan. | Holotype and 3 paratypes |
| *Seira metarsiosa* Coates, 1968 |  | NC | 2 paratypes from lucerne, Kakamas, 2, Dec 1965, M.K.P. Meyer leg, AcV65/274 | 2 paratypes, NIC |
| *Seira metarsiosa* Coates, 1968 |  | NC | Groblersdal. |  |
| *Seira munroi (*Paclt, 1959) | Paclt 1959 | NC | Type locality: Kalahari Gemsbok National Park, Tweede Rivieren, in ants' nests, 12-20.2.1958, H.K. Munro leg. | 1 holotype and 24 paratypes NIC, 7 paratypes Museum Lund, 20 paratypes in author's collection |
| *Seira nagatai* Yosii, 1959 | Yosii 1959 | WC | Type locality: Kloof Nek (2 specimens) 9.xii.1957, Kirstenbosh (2 specimens) 8.xii.1957, Skeleton Gorge (10 specimens) 10.iii.1958. |  |
| *Seira pallens* (Börner, 1908) | Börner 1908 | NC | Steinkopf, Little Namaland, Spring 1904, 20 young and adult specimens, L. Schultze leg. |  |
| *Seira pseudocoerulea* (Denis, 1924) | Womersley 1934 | WC | Franch Hoek 2000 feet Dec 1916 (K.H. Barnard leg.), Kirstenbosch 23.vii.1930, Hout Bay August 1930, Signal Hill, Cape Town, 31.viii.1930 (all Womersley leg.) |  |
| *Seira pseudocoerulea* (Denis, 1924) | Yosii 1959 | WC | Skeleton Gorge, 5 specimens, 11.iii.1958. | . |
| *Seira rowani* Yosii, 1959 | Coates 1968 | WC | Tsitsikama National park, T.J. Coates leg., 4 specimens. |  |
| *Seira rowani* Yosii, 1959 | Coates 1970 | WC | Tsitsikama Forest and Coastal National Park, on *Passerina* sp and on unidentified shrub, Storms River Mouth. |  |
| *Seira rowani* Yosii, 1959 | Yosii 1959 | WC | Top of Table Mountain, 18 specimens, 11.iii.1958. |  |
| *Seira rykei* Coates, 1968 | Coates 1968 | WC | Holotype and 1 paratype from *Metalasia muricata* Less., Robinson Pass, 25.i.1965, AcV65/16; 2 specimens from *Helichrysum crispum* Less., Grootvadersbosch and one from grass, Suurbraak, 11.ii.1965, AcV66/187 & 189 (all T.J.Coates leg). | Holotype, NIC |
| *Seira squamoornata* (Scherbakov, 1898) | Paclt 1959 | FS | Bloemfontein, under a few pine trees, from soil containing large amount of organic plant material, especially fallen needles of pine trees, April 1957, R. VAN PLETZEN leg. (2 specimens). |  |
| *Seira squamoornata* (Scherbakov, 1898) | Paclt 1959 | G | Northen Transvaal, Forst Entabeni, Zoutpansberg Mountains (etwa 40 km ostl. Louis Trichardt). ca. 1500 m, aus Fall-Laub von verschiedenen durcheinander wachsenden Bauman mittels Trichter-Falle im Labor. in Pretoria gcwonnen. Mai 1957 (108)", R. STRASSEN leg. (1 specimen, SMF 1381'). |  |
| *Seira squamoornata* (Scherbakov, 1898) | Paclt 1967 | G | Pretoria, Parktown, grass on banks of Apies River, 24.ix.1937, 1 specimen AcV45, D.v.V. Webb; Rust de Winter, Farm Rooikop, in fungus comb of nest of *Odontotermes transvaalensis* (Sjbst), 20.vi.1963, one specimen T.I07, J. L. Sheasby. | NIC |
| *Seira squamoornata* (Scherbakov, 1898) | Paclt 1959 | KZN | Howick Falls, 9.iii.1905, I. TRAGARDH leg (1 specimen) National Park, Tugela Valley, under stone on fairly wet meadow, alt. about 5000 ft., 3.iv.1951. SSAE leg., loc. 258 (1 specimen) -Albert Falls, in ants' nests, May 1942, R. F. LAWRENCE leg. (1 specimen). - Champagne Castle in the Drakensbergen, February 1946, R. F. Lawrence leg. (25 specimens). – Mont-aux-Sources in the Drakensbergen, alt. 10,500 ft., March 1946, R. F. Lawrence leg. (5 specimens). - Pietermaritzburg, from dry leaves in garden, September 1951, R. F. LAWRENCE leg. (2 specimens). - Champagne Castle in the Drakensbergen, Hotel, January 1951, R. F. Lawrence leg. (4+11 specimens). - Basutoland: Makheke Mountains, 15 miles ENE of Mokhotlong, at stony stream in alpine mountain valley, alt. 9500 ft., 7.iv.1951, SSAE leg, loc. 268 (1 specimen), Hluhluwe Game Reserve, swept among grass, 17.iv.1951, SSAE leg., loc. 276 (1 specimen). | Museum Lund |
| *Seira squamoornata* (Scherbakov, 1898) | Paclt 1967 | KZN | Nongoma, grass sweepings, 15.ix.l922, 28 specimens AcV1; Ingwavuma, grass sweepings, 18.ix.1922, 51 specimens AcV2; all material J. C. Faure leg.; Cedara 11-26.xii.1937, 86 specimens AcV54; Estcourt District, Upper Luteni River, altitude of ± 5000 ft at foot of main Drakensberg mountain range, July 1941, 13 specimens AcV74, 11 specimens AcV75,. 18 specimens AcV76, II specimens AcV77 and II specimens AcV78; Estcourt District, Cathkin Peak, Champagne Castle Hostel, at altitude of ± 6000 ft on slopes of main Drakensberg mountain range, July 1942, 17 specimens AcV79; Mkuzi Game Reserve, grass sweepings, 2I.x.1945, 17 specimens AcV80, light traps, December 1945-January 1946, 5 + 160 specimens AcV81, 1650 specimens; AcV82, and May 1947,176 specimens AcV83; Idem, Umboneni Pan, damp soil, 25.v.1947, 62 specimens AcV84 and Uyenya Pan, 21.ix.1947, six specimens AcV86; Mtubatuba, Duku Duku Forest, 23.ix.1947, 7 specimens AcV87; all material D.v.V. Webb leg. | NIC |
| *Seira squamoornata* (Scherbakov, 1898) | Paclt 1967 | NC | Keimoes, banks of Orange River, December 1937, one specimen AcV46 and 21 specimens AcV50; Upington, banks of Orange River, December 1937, 9 specimens AcV47, 13 specimens AcV48, 8 specimens AcV49, 13 specimens AcV51 and 5 specimens AcV53; Steytlerville, banks of Sundays River, 5.x.1938, 10 specimens AcV56; Uitenhage, Prickly Pear Laboratory, 17.x.1938, 6 specimens AcV57 and Gubb Location, 17.x.1938, 8 specimens AcV58; Glenconner, 28.x.1938, 3 specimens AcV59; Uitenhage, Prickly Laboratory, 2.xi.1938, 7 specimens AcV60, 3.xi.l938, 18 specimens AcV61 and in Wardian cases containing rotted *Opuntia* pads, 4.iv.1939, 16 specimens AcV62; Aberdeen, 2 I.ix. 1939, 14 specimens AcV69; Graaff-Reinet 23.ix.1939, 12 specimens AcV70, 25.ix.1939, 3 specimens AcV72 and 29.ix.1939, 13 specimens AcV73. | NIC |
| *Seira squamoornata* (Scherbakov, 1898) | Paclt 1959 | WC | Yzerfontein, under succulents on sandy beach (fig. 25), 25.x.1950, SSAE leg., loc. 17 (1 specimen). - Franschhoek Bosreserve: Upper Berg River at the stream, alt. 1500 ft., 1.xi. 1950, SSAE leg., loc. 21 (1 specimen) - Cape Peninsula, Cape Point, Nature Reserve, sieved from wet leaves in low bush, 10.xii.1950, SSAE leg., loc. 79 (2 specimens). – Cape Peninsula, Hout Bay, Skoorsteenkop, in insect trap, ~22.xii1950, loc. 78 (I specimen) - 7 miles SW of Bredasdorp in shallow cave in limestone hill about 2 yards from the opening of the cave, 30.xii.19, SSAE leg., loc. 99 (1 specimen) - Ibidem, under stones and among vegetable debris on limestone hill, covered by bush, 30.xii.1950, SSAE leg., loc. 99 (I specimen) - Addo, in garden, 5.i.1951, SSAE leg., loc. a2 (1 specimen) - Cape Peninsula, Hout Bay. Skoorsteenkop, in insect trap, alt. 650 ft., 22-28.i. 1951, SSAE leg. cf. loc. 157 (12 specimens) – Ibidem, sieved from vegetable debris in dense indigenous forest, alt. 600 ft., 28.i. I951, SSAE leg., loc. 161 (1 specimen). | All specimens Museum Lund |
| *Seira squamoornata* (Scherbakov, 1898) | Paclt 1967 | WC | Gordons Bay, Steenbras River mouth, moss and rotten leaves, I.viii.1937, 5 specimens AcV31; Gordons Bay, grass near sea, 4.viii.1937, 2 specimens AcV32; Somerset West, 4.viii.1937, three specimens AcV33; Gordons Bay, 4.viii.1937, 12 specimens AcV34 and 9.viii.1937, 5 specimens AcV35; Strand, 02.viii.1937, 16 specimens AcV39; GordonsBay, 02.viii.l937, 17 specimens AcV40; Somerset West, Sir Lowrys Pass, 12.viii.1937, 7 specimens AcV41; Gordons Bay, 13.VIII.1937, 10 specimens AcV42; Palmiet River, near Kleinmond, 17.viii .193 7, 17 specimens AcV44 ; Gordons Bay, 5.viii.1938, 8 specimens AcV55; Velddrift, damp soil on banks of Berg River 20.iii.1956, 2 specimens AcV90; Langebaan,Oesterwal, grass sweepings along lagoon, 20.iii.1956, 19 specimens AcV91; Citrusdal, banks of Olifants River 15 miles on Clanwilliam road, 21.iii.1956, three specimens AcV92; Clanwilliam, 8 miles on van Rhynsdorp road, 22.iii.l956, three specimens AcV94; Baineskloof Pass, along mountain stream 7 miles from Wellington, 23.iii.1956, 6 specimens AcV96; Calvinia, 120 miles on Ceres road, 23.iii.1956, 6 specimens AcV97; Muizenberg, Boyes Drive Waterfall, damp soil and moss, 15.iv.1956, 2 specimens AcV98; all material D.v.V. Webb leg. | NIC |
| *Seira tsikama* Coates, 1968 | Coates 1968, 1970 | WC | Holotype under oak leaves, Tsitsikama Seacoast National Park, 19.i.1967 (T.J. Coates leg), AcV65/96; paratype from rotting leaves Storms River Mouth, 20.i.1967 T.J. Coates leg, AcV65/104; 1 paratype from indigenous forest litter, Knysna, 9.iv.1966 (J. Findlay leg) aVc66/136. | Holotype and 2 paratypes, NIC |
| *Seira vaneedeni* Coates, 1968 | Coates 1968 | KZN | Holotype from grass, Oribi Gorge, 2.ii.1966 AcV66/116; 1 paratype from unidentified shrub, Charters Creek, St. lucia, 24.i.1966 AcV66/63; 3 paratypes from grass, Mkuze, 24.i.1966 AcV66/76; 1 paratype from grass Park Rynie, 31.i.1966 AcV66/141, about 100 other specimens from the Natal coast, all T.J. Coates leg. | Holotype and 4 paratypes, NIC |
| *Seira* sp. 1 | Liu et al. | WC | Pine and Cape Flats Sand Fynbos litter, Tokai, July/August 2010 |  |
| *Seira* sp. 2 | Liu et al. | WC | Pine and Cape Flats Sand Fynbos litter, Tokai, July/August 2010 |  |
| *Seira* sp. 3 | Liu et al. | WC | Pine and Cape Flats Sand Fynbos litter, Tokai, July/August 2010 |  |
| *Seira* sp. 4 | Liu et al. | WC | Pine and Cape Flats Sand Fynbos litter, Tokai, July/August 2010 |  |
| *Seira* sp. 5 | Liu et al. | WC | Pine and Cape Flats Sand Fynbos litter, Tokai, July/August 2010 |  |
| **Cyphoderidae** |  |  |  |  |
| *Calobatinus rhadinopus* (Börner, 1913) | Paclt 1967 | G | 10 miles East of Pretoria, shelving and fungus comb in nest of *Macrotermes natalensis,* 25.ii.1963, 3 ♂ and 5 ♀ T.23; Rust de Winter, Farm Rooikop, fungus comb in nest of *M. waterbergi* 13.iii.1963, 13 ♀ T.38, all J.S. Sheasby leg. | NIC |
| *Calobatinus rhadinopus* (Börner, 1913) | Börner 1913 | KZN | 4 specimens (3 ♀, 1♂), nest of *Macrotermes natalensis*, 23.vi.1898, no specific location given, Dr. J. Trägardhs leg. |  |
| *Cyphoda colura* (Börner, 1908) | Börner 1908 | NC | Steinkopf, nest of termite *Hodotermes viator,* July 1904, 3 specimens, L. Schultze leg. |  |
| *Cyphoda limboxiphia* (Börner, 1913) | Paclt 1967 | G | 3.6 miles from Pretoria on Warmbaths Road, shelving and fungus comb in cabity of *Odontotermes transvaalensis,* 11.ii.1963, 1 specimen T.2, 4 specimens t.4, 18.ii.1963, 1 specimen t.14 and 8.iii.193, 56 specimens T.34; Rust de Winter, Farm Rooikop, fungus comb in nest of *O. badius,* 19.iii.1963, 12 specimens T.50, all material J.L. Sheasby leg. |  |
| *Cyphoda limboxiphia* (Börner, 1913) | Börner 1913 | KZN | Nest of termite *Trinervitermes trinervius* |  |
| *Cyphoda natalensis* (Börner, 1913) | Börner 1913 | KZN | Nest of termite *Macrotermes natalensis* |  |
| *Cyphoda natalensis* (Börner, 1913) | Womersley 1934 | WC | Ants’ nests, Table Mountain, Cape Town, 27 July 1930, Hout Bay Aug 1930 (Womersley leg). | Two of these specimens observed by Paclt 1959 in SAMC |
| *Cyphoderus assimilis* Börner, 1906 | Paclt 1959 | KZN | Albert Falls, in ants' nests, May 1942, R.F. Lawrence leg. (7 specimens) |  |
| *Cyphoderus assimilis* Börner, 1906 (cf.) | Paclt 1967 | KZN | Van Rhynsdorp, top of van Rhyns pass, 26 miles from van Rhynsdorp, under stones, 22.iii.1956, 2 specimens, AcV95, D.v.V. Webb leg (specimens immature and damaged) | NIC |
| *Cyphoderus bidenticulatus* Parona, 1888 | Börner 1913 | KZN | Nest of *Odontotermes latericius* |  |
| *Cyphoderus omoensis* Delamare Deboutteville, 1945 | Paclt 1959 | WC | On stalagmites (according to slide) Cango Caves, Oudtshoorn, Nov. 1929, K.H. Barnard leg. | SAMC |
| *Cyphoderus squamidives* Silvestri, 1918 | Silvestri 1918 | G | Pretoria, together with *Calobatinus rhadinopus*, with termites *Macrotermes natalensis.* |  |
| *Cyphoderus squamidives* Silvestri, 1918 | Paclt 1959 | KZN | Champagne Castle, Drakensberg, Feb 1946, R. F. Lawrence leg (2 specimens) |  |
| *Cyphoderus squamidives* Silvestri, 1918 | Paclt 1967 | WC | Simons Town, Termite Research Laboratory of the Division of Entomology, Simons Town, soil in colony of *Coptotermes formosanus* 9.viii.1956, 69 specimens, AcV107, Mrs P.E. de Wet leg. | NIC |
| *Cyphoderus trinervoidis* Paclt, 1965 | Paclt 1965 | G | 18 miles from Pretoria in the direction towards Babsfontein, in nest of *Trinervitermes trinervoides* (Sjöstedt), J. L. Sheasby leg. 27.ii.1963 | Holotype SMF A 1677 and 14 paratypes SMF A 1678, 17 paratypes in Entomol. Avdeln. Zool. Inst. Univ. Lund, 12 paratypes in NIC, Locality T.27 |
| *Pseudocyphoderus wasmanni* Börner, 1913 | Paclt 1967 | G | 35.6 miles from Pretoria on the Warmbaths road, shelving and fungus comb in nest of *Odototermes transvaalensis* 11.ii. 1963, 6 specimens T.4; Waverley, 7 miles east of Pretoria, shelving and fungus comb of *O. latericius*, 20,ii.1963, 11 specimens T.21; near Vlakfontein 10 miles east of Pretoria, shelving and fungus comb of *Macrotermes natalenis*, 25.ii.1963, 46 specimens T. 23, all J.S. Sheasby leg. |  |
| *Pseudocyphoderus wasmanni* Börner, 1913 | Börner, 1913 | KZN | Nests of termite *Odontotermes vulgaris*, November 1898, no specific location given. |  |
| **Paronellidae** |  |  |  |  |
| *Dicranocentruga nigromaculata* Schött, 1903 | Paclt 1959 | KZN | Champagne Castle, Drakensberg, Feb 1946, R.F. Lawrence leg, 25 specimens. |  |
| **Tomoceridae** |  |  |  |  |
| *Neophorella dubia* Womersley, 1934* | Womersley 1934 | WC | Table Mountain, Cape Town, 2500 feet, 12.ix.1913, K.H. Barnard leg | 1 specimen SAMC, slide in bad condition. |
| *Tomocerus minor* (Lubbock, 1862) (cf.) | Liu et al. 2012 | WC | Pine litter, Tokai, July/August 2010 |  |
| **NEELIPLEONA** |  |  |  |  |
| **Neelidae** |  |  |  |  |
| *Megalothorax minimus* (Willem, 1900) | Paclt 1967 | WC | Hout Bay, Chapman’s Peak Drive, damp soil and moss, 15.iv.1956, 16 specimens, AcV99; Table Mountain Drive Waterfall No. 2, damp moss, 5.v. 1956, 6 specimens, AcV106, all D.v.V. Webb leg. | NIC |
| *Megalothorax* sp. | Liu et al. 2012 | WC | Pine and Cape Flats Sand Fynbos litter, Tokai, July/August 2010 |  |
| *Neelus* sp. | Liu et al. 2012 | WC | Pine litter, Tokai, July/August 2010 |  |
| **SYMPHYPLEONA** |  |  |  |  |
| **Mackenziellidae** |  |  |  |  |
| *Mackenziella psocoides Hammer, 1953* (cf.) | Liu et al. 2012 | WC | Cape Flats Sand Fynbos litter, Tokai, July/August 2010 |  |
| **Sminthurididae** |  |  |  |  |
| *Denisiella serroseta* Börner, 1908 | Börner 1908 | NC | Kalahari, between the pans Sekgoma and Khakhea, November 1904, L. Schultze leg, ♂ very numerous, ♂ and ♀, found in thick clusters, L. Schultze leg. |  |
| *Sminthurides* sp. 1 | Liu et al. 2012 | WC | Cape Flats Sand Fynbos litter, Tokai, July/August 2010 |  |
| *Sminthurides* sp. 2 | Liu et al. 2012 | WC | Pine and Cape Flats Sand Fynbos litter, Tokai, July/August 2010 |  |
| *Sphaeridia minimus* (Schött, 1893) | Paclt 1959 | FS | Bloemfontein, garden, from soil containing large amount of organic plant material, August 1957, R. van Pletzen, 8 specimens. |  |
| *Sphaeridia minimus* (Schött, 1893) | Paclt 1967 | WC | van Rhynsdorp, Knersvlakte, 3 miles from van Rhyns pass, 22.iii.1956, , 1 ♂and 6 ♀, AcV93; Simons Town, Red hill Drive, damp soil and moss, 22.iv.1956, 2 ♀ AcV 101; Table Mountain Drive, humus and pine needles, 5.v.1956, 1 ♀ AcV105, all D.v.V. Webb leg. | NIC |
| *Sphaeridia* sp. | Liu et al. 2012 | WC | Pine and Cape Flats Sand Fynbos litter, Tokai, July/August 2010 |  |
| **Arrhopalitidae** |  |  |  |  |
| *Arrhopalites* sp. | Liu et al. 2012 | WC | Pine litter, Tokai, July/August 2010 |  |
| **Katiannidae** |  |  |  |  |
| *Katianna kerguelenensis* Denis, 1947 | Paclt 1959 | KZN | Champagne Castle, Drakensberg, R. F. Lawrence leg, Feb 1946, 1 specimen. | Paclt found 1 specimen in vial in SAMC from Womersley collection, maybe from SW Cape or S. Zimbabwe. |
| *Sminthurinus elegans* (Fitch, 1862) (cf.) | Liu et al. 2012 | WC | Pine litter, Tokai, July/August 2010 |  |
| *Sminthurinus mime* (Börner, 1907) | Paclt 1967 | WC | Paarl, fallen leaves, 29.viii.1937, 1 specimen, AcV29; Gordon’s Bay 9.viii.1937, 27 specimens AcV37, 11.vii.1937, 1 specimen AcV43; Strand, 11.viii.1937, 3 specimens AcV39, all D.v.V. Webb leg. | NIC |
| *Sminthurinus mime* (Börner, 1907) | Womersley 1931, same citation in Paclt 1959 | WC | Stellenbosch, surface of ground beneath strong grow of *Cryptostemma calendulaceum* (Cape weed) in Stellenbosch University orchard, 24.vii.1930 and 22-24.viii.30; similar habitat Marsh Memorial Homes, Rondebosch, August 1930. | Type in SAMC |
| *Sminthurinus niger* (Lubbock, 1873) | Paclt 1959 | WC | Stellenbosch | One specimen in SAMC |
| *Sminthurinus niger* (Lubbock, 1873) | Womersley 1931 | WC | Stellenbosch, under loose bark of fallen log, C.P., 12.viii.1930. |  |
| *Sminthurinus pallidus* Womersley, 1931 | Womersley 1931, same citation in Paclt 1959 | WC | Type locality: Stellenbosch, surface of ground beneath strong growth of *Cryptostemma calendulaceum* (Cape weed) in Stellenbosch University orchard, 28.9.1930 | Holotype in SAMC (a single specimen) |
| *Sminthurinus* sp. 1 | Liu et al. 2012 | WC | Pine and Cape Flats Sand Fynbos litter, Tokai, July/August 2010 |  |
| *Sminthurinus* sp. 2 | Liu et al. 2012 | WC | Pine and Cape Flats Sand Fynbos litter, Tokai, July/August 2010 |  |
| *Sminthurinus* sp. 3 | Liu et al. 2012 | WC | Pine litter, Tokai, July/August 2010 |  |
| *Stenognathellus stenognathus* (Börner, 1907) | Paclt 1959 | KZN | Pietermaritzburg, dry leaves in garden, R.F. Lawrence Sept 1951, 61 specimens. |  |
| *Stenognathellus stenognathus* (Börner, 1907) | Paclt 1959 | WC | Franschhoek Forest Reserve, Upper Berg river, at stream, alt 1500 ft., 1.xi.1950, SSAE leg, loc 21 (1 specimen), Cape Peninsula, Cape Point Nature Reserve, sieved from wet leaves in low bush, 2400 ft., 18.xii.1950 Loc. 84 (9 specimens), Hout Bay, Skoorsteenkop, sieved from vegetable debris in dense indigenous forest, 600 ft., 28.i.1951, loc. 161 (14 specimens), all SSAE leg. | Museum Lund |
| **Dicyrtomidae** |  |  |  |  |
| *Dicyrtomina africana* Womersley, 1931 | Womersley 1931 | WC | Elsenburg, on native olive bushes, 24.vii.1930, 28.viii.1927 leg Dr. Hesse. |  |
| *Dicyrtomina minuta* O. Fabricius, 1783 | Paclt 1959 | WC | Kirstenbosch, at stony stream, shaded by dense vegetation, 6.vii.1951, SSAE leg. Loc. 352. | 4 specimens Museum Lund |
| *Dicyrtomina minuta* O. Fabricius, 1783 | Paclt 1967 | WC | Somerset West, 4.viii.1937, 26 specimens AcV33; Gordon’s Bay, 10.viii.1937, 20 specimens AcV38; Palmiet River near Kleinmond 17.viii.1937, 5 specimens AcV44; Gordon’s Bay 5.viii.1938, 1 specimen AcV55, all D.v.V. Webb leg. | NIC |
| *Dicyrtomina ornata* (Nicolet, 1842) (cf.) | Liu et al. 2012 | WC | Pine and Cape Flats Sand Fynbos litter, Tokai, July/August 2010 |  |
| *Dicyrtomina saundersi* (Lubbock, 1862) (cf.) | Liu et al. 2012 | WC | Pine and Cape Flats Sand Fynbos litter, Tokai, July/August 2010 |  |
| *Dicyrtomina* sp. | Liu et al. 2012 | WC | Cape Flats Sand Fynbos litter, Tokai, July/August 2010 |  |
| **Bourletiellidae** |  |  |  |  |
| *Bourletiella arvalis* (Fitch, 1863) | Paclt 1959 | WC | Outeniqua Experimental Farm, George, on lucerne pasture, 15.9.1958, J. J. C. Nel leg. | 3 ♂ and 15 ♀specimens, Dept. Agric. Stellenbosch; 4 ♂ and 25 ♀ specimens in Paclt’s collection |
| *Bourletiella* sp. | Liu et al. 2012 | WC | Cape Flats Sand Fynbos litter, Tokai, July/August 2010 |  |
| *Prorastriopes barnardi* (Womersley, 1931) | Paclt 1959 | WC | Hangklip, 14 miles S of Strand, under stone on dry *Juncus* heath, 19.xii.1950, SSAE leg, loc. 86. Maanschijnkop (Maanskynkop?), 7 miles E of Hermanus, swept in dry vegetation, heath, 21.12.1950, SSAE leg. Loc. 93. | 15 ♀ specimens, Museum Lund |
| *Prorastriopes barnardi* (Womersley, 1931) | Womersley 1931 | WC | Hottentots Holland Mountains, Jan 1916, K.H. Barnard leg., and top of Kalk Bay Mountain, Cape Peninsula, amongst grass, 12.i.1912. | Co-types in SAMC |
| *Prorastriopes marmoratus* (Womersley, 1931) | Womersley 1931 | WC | Hottentots Holland Mountains, Jan 1916, K.H. Barnard leg., rain pools at 4000 ft.; and top of Kalk Bay Mountain, Cape Peninsula, 12.i.1912. | Co-types in SAMC |
| *Prorastriopes schultzei* (Börner, 1908) | Börner 1908 | NC | Steinkopf and Kamaggas, Spring 1904, L. Schultze leg. |  |
| *Prorastriopes webbi* Paclt, 1964 | Coates 1970 | EC | Mountain Zebra National park, on *Diospyros austrafricana* var. *microphylla* and on unidentified shrub on top of mountain near beacon. |  |
| *Prorastriopes webbi* Paclt, 1964 | Paclt 1964 | KZN | Mkuzi Game Reserve, Zululand, found at light, D. v. V. Webb leg, December 1945 to January 1946. | Holotype ♀, SMF A 1673, Paratypes 2 ♀, NIC AcV 82, Natural History Museum, Entomology Section, Frankfurt |
| *Prorastriopes webbi* Paclt, 1964 | Coates 1970 | MP | Kruger National Park, under rotting leaves of *Bougainvillea*xsp. Shingwidzi Rest Camp |  |
| *Rastriopes lineata* Womersley, 1931 | Paclt 1967 | G | Pretoria, Parktown, grass on banks of Apies River, 24.ix.1937, 2 specimens, AcV45, D.v.V. Webb leg | NIC |
| *Rastriopes lineata* Womersley, 1931 | Paclt 1967 | NC | Graaff-Reinet 29.ix.1939, 6 specimens AcV73, D.v.V. Webb leg. | NIC |
| *Rastriopes lineata* Womersley, 1931 | Paclt 1959 | WC | Table Mountain, swept among vegetation, Juncus tufts, alt 3400 ft., 7.xii.1951, SSAE leg, loc.353. | 1 ♀ specimen, Museum Lund |
| *Rastriopes lineata* Womersley, 1931 | Paclt 1967 | WC | Mamre Road, Waterkloof Farm, 3.vi.1937, 1 specimen AcV28; Gordons Bay, Steenbras River Mouth, moss and rotten leaves 1.viii.1937, 1 specimens AcV31, Gordons Bay, 4.viii.1937, 2 specimens AcV34, 1 specimen AcV 40 and 13.viii.1937, 2 specimensAcV42, Palmiet River near Kleinmond, 17,viii,1937, 1 specimen, AcV44, all D.v.V. Webb leg. | NIC |
| *Rastriopes lineata* Womersley, 1931 | Womersley 1931 | WC | Under fallen twig. Kloof Nek, Cape Town, 27.vii.1930. Also 4 immature specimens from surface of rain pools in Cape Town, K.H. Bernard leg, 09.v.1916. | Holotype in SAMC |
| *Tritosminthurus schuhi* Snider, 1988* | Snider 1988 | WC | Cape Province, Cape Point Nature Reserve, 30.i.1968, R.T. Schuh, J. and S. Slater and M. Sweet legs. | Holotype ♀, 100+ co-types, 31 slides, all deposited in Entomology Museum, Michigan State University |
| **Sminthuridae** |  |  |  |  |
| *Papirinus prodigiosum* Yosii, 1954 | Paclt 1959 | KZN | Champagne Castle, Drakensberg, Feb 1946, R.F. Lawrence leg. (1 specimen). |  |
| *Sminthurus viridis* (Linnaeus, 1758) | Lawrence 1953 | ? | No information given. |  |
| *Sminthurus viridis* (Linnaeus, 1758) | Paclt 1959 | WC | Somerset West, on oats, July 1941, Mally leg., Caledon, on Lucerne pastures, August 1956, Dept. Agric Stellenbosch leg. | Somerset West: 2 ♀ and 11 ♀ specimens in Dept. Agric. Stellenbosch, 11 ♀ specimens in author's collection, Caledon: 2 ♀ and 2 ♀ specimens at Dept. Agric. Stellenbosch, 16 ♀ and 20 ♀ specimens in author's collection. |
